# Multi-Task Cascade Forest Framework for Predicting Acute Toxicity across Species

**DOI:** 10.34133/research.1046

**Published:** 2026-01-15

**Authors:** Kunhong Liu, Ruijiang Li, Lianlian Wu, Jun Yang, Junshan Han, Song He, Xiaochen Bo, Jie Gao

**Affiliations:** ^1^ Department of Digital Media, School of Film, Xiamen University, Xiamen 361005, China.; ^2^ Xiamen Key Laboratory of Intelligent Storage and Computing, School of Informatics, Xiamen University, Xiamen 361005, China.; ^3^ Department of Advanced & Interdisciplinary Biotechnology, Academy of Military Medical Sciences, Beijing 100850, China.; ^4^ Academy of Medical Engineering and Translational Medicine, Tianjin University, Tianjin 300072, China.; ^5^ Department of Cell Biology, School of Life Sciences, Central South University, Changsha 410013, China.; ^6^ Department of Epidemiology and Health Statistics, School of Public Health, Fujian Medical University, Fuzhou 350122, China.

## Abstract

Evaluating chemical toxicity and its potential hazards to human health and the environment is essential in diverse fields, including medicine, industry, and agriculture. Multi-species acute toxicity prediction (MSATP) is critical in toxicity assessment. Traditional methods rely on exposing animals to a single high dose of a compound and observing its toxicity. However, with growing ethical concerns regarding animal testing, advancements in computational technology have positioned the artificial intelligence-based MSATP as an efficient alternative. Current research on MSATP commonly employs multi-task deep neural networks for modeling. However, the small size, high dimensionality, and sparsity of MSATP tabular data render them unsuitable for neural network approaches. To address this, we proposed a multi-task cascade forest framework for MSATP. This framework integrated feature enhancement through knowledge transfer, sample enhancement using a greedy search strategy with the covariance distance measure. The framework accommodated tasks of varying sizes in multi-task learning and was specifically designed for tabular data, achieving a 12% improvement in performance compared to current state-of-the-art methods (*R*^2^ equals to 0.64, root mean square error equals to 0.57). In a single-view context, we conducted ablation experiments to validate the effectiveness of the data enhancement strategy and introduced external dataset experiments to assess the generalization capability of the proposed method for cross-species prediction. In a multi-view context, the feature fusion method and consensus ensemble were demonstrated to further enhance the model performance. Additionally, we analyzed feature importance vectors to provide interpretable insights into species toxicity correlations. Overall, this framework effectively addressed MSATP tasks and exhibited substantial potential for application in various toxicity prediction domains.

## Introduction

Chemical toxicity evaluation is vital in the medical, industrial, and agricultural sectors to ensure rigorous safety testing and to prevent harmful effects on the environment, living organisms, and humans [1–3]. During drug discovery and development, identifying compounds with the highest potential for safety and efficacy can reduce failure rates during early design stages [4–6]. Multi-species acute toxicity prediction (MSATP) is a critical yet challenging aspect of toxicity evaluation. Traditional MSATP methods rely on in vivo animal studies combined with in vitro techniques, which are labor-intensive, costly, and time-consuming. Moreover, the extensive use of laboratory animals has raised significant ethical concerns worldwide [7,8]. With the growing availability of MSATP data, machine learning has emerged as a cost-effective, rapid, and precise alternative that offers an efficient solution to reduce reliance on animal testing [9–11].

Jain et al. [5,11] conducted diverse acute toxicity measurements across various species and administration modes, compiling a comprehensive MSATP dataset comprising 122,594 measurements, 80,081 compounds, and 59 toxicity endpoints. The publication of this dataset has significantly advanced MSATP domain modeling, with detailed studies extensively reporting its utilization [12]. The dataset represents typical multi-task, multi-view data formally defined in machine learning terms. In a multi-task single-view context, the dataset contains 59 prediction tasks, each represented as $D_{i}=\left\{\left.\left(X_{i},Y_{i}\right)\;\right|\;i\in \left[1,\;59\right]\right\}$, where $X_{i}\in R^{n_{i}\times d},$ $n_{i}$ is the dataset size, *d* is the sample dimension, and $Y_{i}$ is the corresponding label vector (continuous values indicate toxicity strength). The dataset sizes vary from small to large ($n_{i}\in \left[102,\;36,\;295\right]$), with a total of 122,594 samples $\left(\sum_{i=1}^{59}n_{i}=122,594\right)$. Each sample represents a compound’s molecular expression as high-dimensional sparse tabular data with numerous non-informative features. Compounds may have different representations across chemical spaces, resulting in varied representation vectors in multi-view contexts. The goal of MSATP modeling is to simultaneously learn all 59 tasks and leverage the knowledge from all related tasks to enhance performance. This can be approached as multi-task single-view or multi-task multi-view learning. Task performance is evaluated using $P_{i}$ for individual tasks and $P_{\mathrm{avg}}=\left(\frac{1}{59}\sum_{i=1}^{59}P_{i}\right)$ for the overall average. The ultimate objective is to maximize $P_{\mathrm{avg}}$ to improve the overall efficacy of multi-task learning in the MSATP domain.

In recent years, many modeling studies have been proposed based on the aforementioned dataset, primarily combining multi-task and multi-view approaches with deep neural networks (DNNs) [9–11]. However, we suggest that DNNs may be unsuitable for modeling high-dimensional, sparse, and small-sample tabular data for the following reasons. (a) DNNs are designed with induced biases to match the invariance and spatial dependence of data. However, such invariance is rarely present in tabular data characterized by heterogeneous features and extreme values. Although some studies have attempted to transform tabular data to fit DNN structures, the results indicate that handling numerous sparse features through transformations can degrade DNN performance because of rotation invariance [13]. (b) DNNs rely on inverse gradient propagation, which favors smooth gradient-dependent differentiable spaces. However, jagged, sharp, and discontinuous nondifferentiable functions often provide a better fit for tabular data, as demonstrated in several studies [14]. (c) The MSATP dataset predominantly comprises small- and medium-sized datasets, making DNNs prone to overfitting when modeling these data. (d) DNNs are black-box models that require post hoc interpretation methods to approximate their prediction behavior. Such explanations often depend heavily on the interpretation method used and fail to fully elucidate the prediction mechanisms for tabular data [15]. These limitations suggest that alternative modeling approaches may be more effective for MSATP data.

In contrast to DNNs, tree-based models effectively address the challenges of modeling high-dimensional, sparse, and small-to-medium-sized tabular datasets. They learn objective functions through decision rules, which better capture sharp and irregular decision boundaries in the tabular data. Additionally, tree-based models are self-explanatory and well-suited for small- and medium-sized datasets. However, MSATP data include both large-scale and small-to-medium-sized datasets, requiring a performance trade-off to balance accuracy across datasets of varying sizes. A single decision tree or a standard ensemble learning model may not adequately handle the computational complexity of large-scale data. To address these limitations, Zhou and Feng [16] proposed a cascaded forest model that combined the strengths of deep learning, such as layer-by-layer data processing, in-model feature transformation, and sufficient complexity, with the advantages of tree-based models, including decision rules, self-explanation, and suitability for tabular data. The cascade forest model demonstrated robust performance on small- and medium-sized tabular datasets and achieved results comparable to those of DNNs on large datasets. Building on this motivation, this study introduced a tree-based deep learning approach as the foundational framework, integrating different knowledge transfer strategies for multi-task learning. It balanced the modeling performance across datasets of varying sizes and proposes a novel cascading forest framework for acute toxicity (CFF-AT) prediction across multiple species.

The execution steps of the proposed algorithm are summarized as follows. (a) The training data from all tasks were aggregated to create a source domain model rich in knowledge. (b) The source domain model enhanced the features of specific task samples, thereby improving their representation in the feature space. (c) The distance matrix among all tasks was constructed using covariance distance to identify similarities between tasks. (d) Guided by the covariance distance, a greedy search strategy identified the optimal dataset for modeling each task. This approach enhanced the feature and sample spaces for each toxicity endpoint, enabling effective MSATP across endpoints of varying sizes and demonstrating clear advantages over the existing methods. In a single-view context, the ablation experiments validated the effectiveness of the data enhancement strategy, whereas the external data experiments confirmed the method’s generalization capability for cross-species prediction. In addition, in a multi-view context, performance can be further improved through feature fusion modeling or consensus ensemble. The analysis of feature importance vectors identified the correlation factors influencing the modeling of different species endpoints. In summary, this framework effectively addressed cross-species acute toxicity prediction, offering scalability and significant potential for toxicology applications.

## Results

### Comparison algorithms and evaluation metrics

A review of multi-task learning categorized the algorithms into 3 main types: feature-based, sample-based, and parameter-based. In the experimental section, the performance of the proposed method was evaluated and compared with 7 benchmark algorithms.

The first 5 methods, MT-DNN [9], MT-GCNN [17], DLCA [10], ConsenA [11], and ConsenB [11], were derived from recently published studies on MSATP modeling. These methods, which utilize DNNs as their foundational models, fall under feature-based multi-task learning. For instance, MT-DNN is a multilayer feedforward neural network that trains on the combined datasets of all tasks with a separate output unit for each toxicity endpoint at the output layer [9]. MT-GCNN is a variant of the graph convolutional neural network and learns molecular representations based on the graph structure of input molecules [17]. DLCA is a deep learning consensus framework that employs multi-view features to train various MT-DNNs, integrating the resulting models through average outputs [10]. Consensus A and consensus B are additional consensus frameworks; consensus A combines MT-DNN and MT-GCNN, whereas consensus B integrates MT-DNN, MT-GCNN, and DLCA [11]. Building on previous research, consensus A and consensus B integrate additional models and apply parameter tuning through a grid search, achieving improved prediction performance by incorporating larger models. Among these methods, DLCA and consensus B utilize multi-view data as input, with consensus B demonstrating superior performance. All 5 methods were designed for multi-task learning and applied to the same dataset used in this study.

Multi-task learning can be viewed as a specialized form of transfer learning. Accordingly, the last 2 comparison methods are adaptations of standard transfer learning algorithms: TranAda [18] and LinInt [19]. Assuming that the training sets for individual tasks are ${\left\{D_{i}\right\}}_{i=1}^{59}$, we treated $D={⋃}_{i=1}^{59}D_{i}$ as the source domain and $D_{i}$ as the target domain. Standard transfer learning algorithms were then adopted to transfer knowledge from D to $D_{i}$ when modeling the *i*th task. TranAda was a supervised instance-based domain adaptation method that follows a “reverse boosting” principle, decreasing the weights of poorly predicted source instances while increasing those of the target instances in each boosting iteration [18]. LinInt is a parameter-based method that interpolates the predictions of the source and target models linearly [19]. The details of the model configurations for both the comparison and the proposed methods are provided in Section A of Supplementary Materials.

This study employed supervised regression, using 2 metrics, coefficient of determination and root mean square error (*R*^2^ and RMSE [11]), to evaluate the model performance [Eqs. (1) and (2)]. In this study, ${\hat{y}}_{i}$ represents the model’s predicted value for unknown samples, $y_{i}$ denotes the true label of unknown samples, ${\bar{y}}_{i}$ is the average label value of all training samples, and *n* is the number of unknown samples. The RMSE measures the error between the model’s output and true labels, with smaller values indicating better performance. The *R*^2^ value typically ranges from 0 to 1, with larger values reflecting a better model fitting. However, it should be noted that in cases of extremely poor performance, the *R*^2^ score can be negative.

$$\text{RMSE}=\sqrt{\frac{1}{n}\;\sum_{i=1}^{n}{\left({\hat{y}}_{i}-y_{i}\right)}^{2}}$$ (1)

$$R^{2}=1-\frac{\sum_{i}^{n}{\left({\hat{y}}_{i}-y_{i}\right)}^{2}}{\sum_{i}^{n}{\left(y_{i}-{\bar{y}}_{i}\right)}^{2}}$$ (2)

### Model performance comparison

Jain et al. [11] compared the modeling performance of various molecular fingerprints, including Avalon, Morgan, and RDKit descriptors, in multi-task deep learning, and reported that models using Avalon molecular fingerprints as inputs achieved the best prediction performance. Consequently, the Avalon molecular fingerprint was selected as the input feature for the main experiments in this study.

Table 1 presents the average performance of the proposed algorithm compared with 7 benchmark algorithms across 59 toxicity datasets. For a single toxicity endpoint, the performance of an algorithm is denoted by ${\left\{P_{i}\right\}}_{i=1}^{59}$, where $P_{i}$ represents the mean result from 10 runs of 5-fold cross-validations. The overall average performance is calculated as $P_{\mathrm{avg}}=\frac{1}{59}\sum_{i=1}^{59}P_{i}$.

**Table 1.** Comparison of average performance of all methods at 59 toxicity endpoints

ID	Model	Input feature type	*R* ^2^	RMSE
1	MT-DNN	Avalon	0.51 _**(5.0)**_	0.69 _**(5.0)**_
2	MT-GCNN	Molecular graph	0.50 _**(6.0)**_	0.70 _**(6.0)**_
3	DLCA	Avalon, Morgan, Atompair, RDKit, SMILES notation	0.54 _**(4.0)**_	0.67 _**(4.0)**_
4	ConsenA	Avalon, Molecular graph	0.55 _**(3.0)**_	0.66 _**(3.0)**_
5	ConsenB	Avalon, Morgan, Atompair, RDKit, SMILES notation	0.57 _**(2.0)**_	0.65 _**(2.0)**_
6	LinInt	Avalon	0.36 _**(7.0)**_	0.78 _**(7.0)**_
7	TranAda	Avalon	0.20 _**(8.0)**_	0.87 _**(8.0)**_
8	CFF-AT	Avalon	0.61 _(1.0)_	0.61 _(1.0)_

Rank sorting was used to interpret the results in Table 1, with values in parentheses indicating the performance ranking of each algorithm under a specific evaluation index, where 1 represents the best performance, 2 represents the second-best performance, and so on. As shown in Table 1, the proposed algorithm CFF-AT achieved the highest rank under both evaluation indicators, followed by ConsenB. Notably, ConsenB integrated multiple DNNs and used multi-view data as the input, whereas CFF-AT relied solely on Avalon single-view data but improved the *R*^2^ performance by 7%. This highlighted the superior average performance of CFF-AT across all toxicity endpoints.

**Fig. 1.**
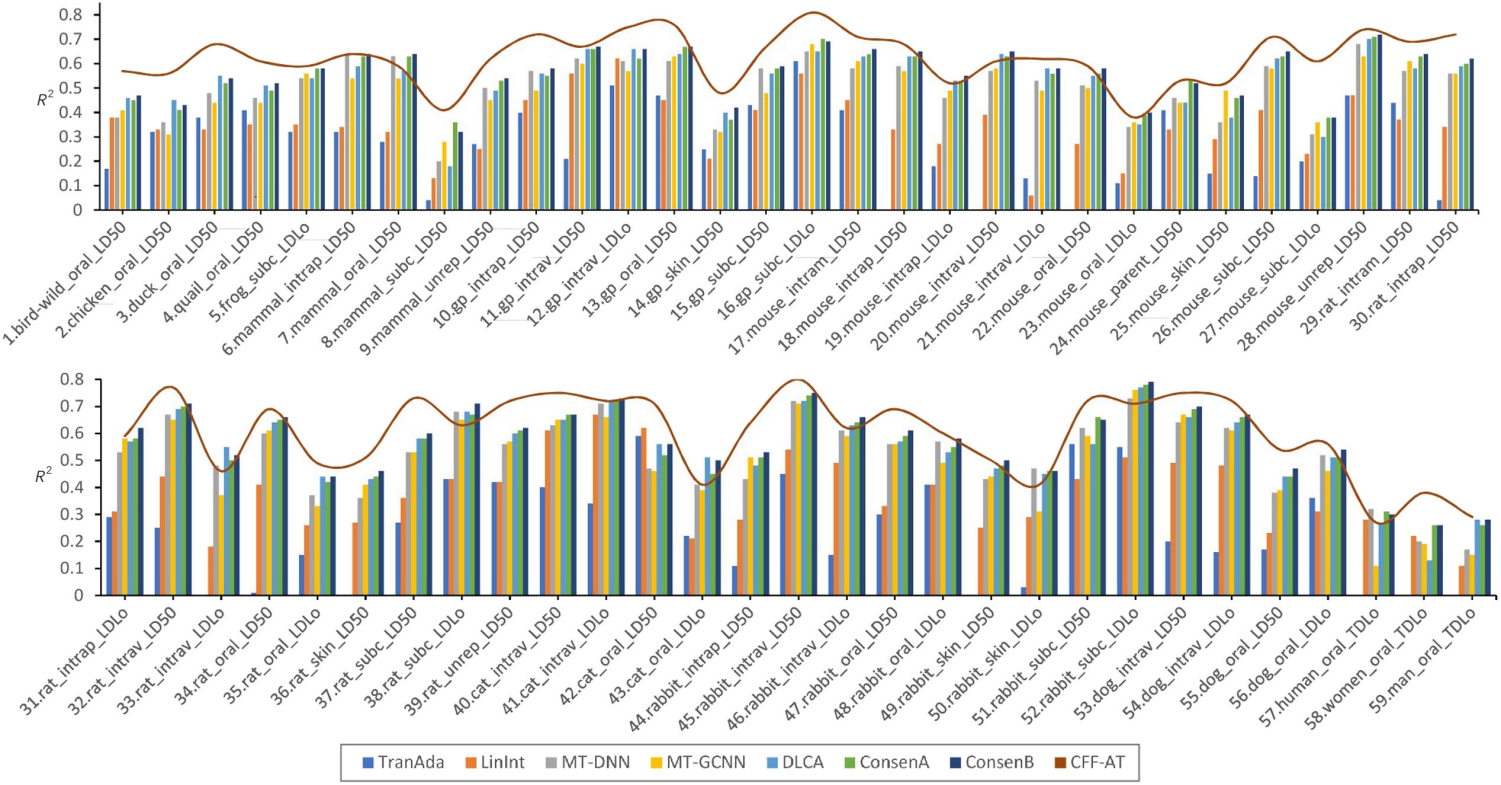
Comparison of *R*^2^ performance of all algorithms at 59 toxicity endpoints.

The detailed performance of all algorithms across the 59 toxicity endpoints is presented below. In Fig. 1, the *y* axis represents *R*^2^, the *x* axis lists the toxicity endpoints, and the histogram for each endpoint shows the performance of the 7 comparison algorithms. The red curve indicates the performance of the proposed method. When the peak or trough of the curve was above the histogram, the proposed algorithm outperformed all comparison algorithms for the toxicity endpoint. The observations revealed that the proposed algorithm generally achieved a superior performance across most toxicity endpoints. Because the RMSE values were minimized, this metric was not suitable for the visual representation in this chart. A detailed table of the performance of all methods on the 59 endpoints is provided in Section B of Supplementary Materials.

**Fig. 2.**
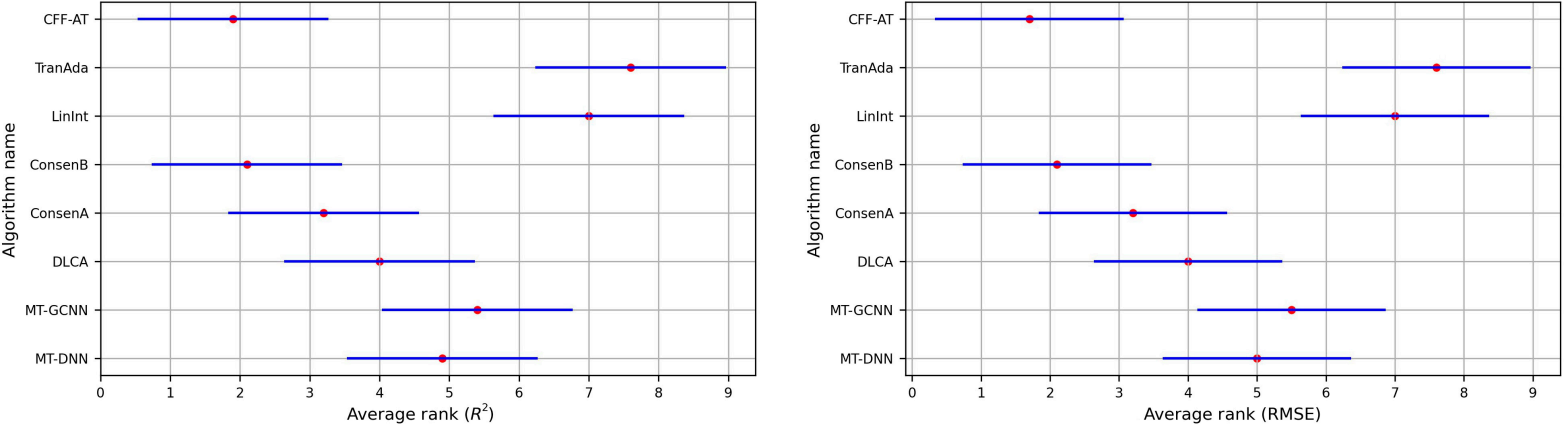
Analysis of algorithm performance ranking difference based on Friedman and Nemenyi tests.

The Friedman test and Nemenyi test [20] were applied to analyze the performance ranking differences among all algorithms across the 59 toxicity endpoints. Detailed results are provided in Section C of Supplementary Materials. Figure 2 visualizes the statistical outcomes, where the *y* axis lists the algorithm names and the *x* axis represents rank sorting values, with smaller values indicating better performance. The red dots in the figure correspond to the rank value of each algorithm, and a horizontal distance exceeding half the straight line between the 2 red dots signifies a significant difference in rank statistics. As shown in Fig. 2, the proposed algorithm demonstrated statistically significant performance differences compared with the other algorithms across all toxicity endpoints, except for the closely ranked ConsenB.

### Ablation experiments under single-view feature

The CFF-AT framework comprised 3 sequential components: source domain cascade forest generation based on multi-task data aggregation, feature enhancement through layer transfer from the source domain model, and sample enhancement using covariance distance measurement and a greedy neighbor search strategy. For clarity, these components are denoted by superscripts 1, 2, and 3, respectively. Different versions of the algorithm were developed to evaluate the performance gains contributed by each component.

Table 2 presents the *R*^2^ performance of the proposed algorithm under different configurations. Model 1 represents a cascade forest in single-task mode, serving as the benchmark for ablation experiments. Model 2 incorporates multi-task data aggregation, improving the performance by 0.05. Building on model 2, model 3 introduced a feature enhancement strategy based on cascade layer transfer, resulting in a performance increase of 0.13. Finally, model 4 adds a sample enhancement strategy to model 3, further improving performance by 0.02. The ablation experimental results demonstrated that all 3 components contributed to enhancing the performance of the cascade forest, with feature enhancement through cascade layer transfer being the most influential.

**Table 2.** Ablation experiments

ID	Model	Description	*R* ^2^	Increase
01	ST-CF	Single-task CF was modeled separately on each TE.	0.41	Baseline
02	CFF-AT^**1**^	Multi-task CF was modeled by data aggregation.	0.46	**↑** 0.05
03	CFF-AT^**12**^	Multi-task CF with feature enhancement.	0.59	**↑** 0.13
04	CFF-AT^**123**^	Multi-task CF with feature/sample enhancement.	0.61	**↑** 0.02

CF, cascade forest; TE, toxicity endpoint

### Generalization ability of the proposed algorithm on external datasets

To evaluate the generalization ability of the CFF-AT model, 3 external datasets were selected: multi-species toxicity in aquatic animals [21], Tetrahymena toxicity [22], and mammalian toxicity in rats [23]. These datasets, categorized as the Avalon type, were characterized by 1,024-dimensional (1,024D) Boolean features.

Table 3 presents the modeling performance of the proposed algorithm on the selected external datasets, where *R*^2^_(CF)_ denotes the performance of the cascade forest model in single-task mode on the external datasets and *R*^2^_(CFF-AT)_ represents the performance of the proposed algorithm in multi-task mode, incorporating knowledge transfer from 59 toxicity endpoints to the external datasets.

**Table 3.** Generalization ability of the proposed algorithm on external validation datasets

External dataset name	Feature type	Sample size	*R* ^2^_(CF)_	*R* ^2^_(CFF-AT)_	Increase
Aquatic animal (multi-species)	Avalon	1,657	0.56	0.60	**↑** 0.04
Aquatic animal (tetrahymena)	Avalon	1,571	0.71	0.74	**↑** 0.03
Mammal (rat)	Avalon	7,385	0.65	0.83	**↑** 0.18

The experimental results demonstrated that the cross-species knowledge transfer capability of the proposed algorithm significantly enhanced the modeling performance on external datasets. The performance of the algorithm on the multi-species aquatic animal dataset (*R*^2^ = 0.60) was comparable to the average performance across the 59 toxicity endpoints (*R*^2^ = 0.61), whereas its performance on the Tetrahymena dataset (*R*^2^ = 0.74) exceeded the average. Furthermore, mammalian rats were among the species included in the 59 toxicity endpoints, whereas aquatic animals were not. It can be inferred that the external rat dataset (*R*^2^ = 0.83) benefited more from knowledge transfer during modeling.

### Introducing multi-view feature and prior knowledge of toxicity endpoints

In the MSATP datasets, the dimensions and values of the same sample vary across different feature types. Table 4 lists the 6 feature types used in this section’s experiments, with the objective of integrating multi-view data to leverage the diverse chemical spaces of samples and improve modeling performance. Additionally, Sosnin et al. [9] highlighted that incorporating prior knowledge of toxicity endpoints into samples enhances the modeling performance. As noted in the previous section, the multi-species acute toxicity data used in this study included 15 species, 8 administration modes, and 3 toxicity types. To emphasize the association between the samples and toxicity endpoints, a 3D vector (*x*, *y*, *z*), where $x\in \left[1,15\right],y\in \left[1,8\right],z\in \left[1,3\right]$, was concatenated with the sample for each endpoint. Both multi-view data and prior knowledge of toxicity endpoints can be viewed as feature space enhancements for sample representation.

**Table 4.** Multi-view feature modeling before and after introducing prior knowledge

Feature type	Feature dimension	Value type	*R*^2^ (raw feature)	*R*^2^ (with prior knowledge)
Avalon	1,024	Boolean	0.61	0.61
AtomPair	1,024	Int	0.60	0.60
Morgan	1,024	Boolean	0.61	0.61
Chemopy	541	Floating	0.47	0.53 **↑**
Unimol	512	Floating	0.33	0.48 **↑**
ChemBERTa	384	Floating	0.06	0.12 **↑**

The results in the last 2 columns of Table 4 indicated that the proposed algorithm performs well on the top 3 feature types, Avalon, AtomPair, and Morgan, whereas they were less influenced by prior knowledge. The introduction of prior knowledge improved the performance of the last 3 feature types (Chemopy, Unimol, and ChemBERTa), but their overall performance remained poor. Based on the value types in Table 4, we hypothesized that prior knowledge of toxicity endpoints would be particularly beneficial for feature types with floating-point values but not for those with Integer or Boolean values. Finally, the top 3 feature types with the best performance were selected for fusion modeling based on multi-view features.

### Exploration of multi-view feature modeling methods

Based on the CFF-AT model, we explored 3 multi-view modeling methods: feature concatenation method [6], feature fusion method [24], and consensus framework [11]. Using the performance of the CFF-AT single-view model (*R*^2^ = 0.61, RMSE = 0.61, with Avalon as input) as the benchmark, we applied Avalon, AtomPair, and Morgan as inputs in the multi-view models. Both the feature fusion method and consensus framework demonstrated performance improvements, with the consensus framework yielding the best performance (*R*^2^ = 0.64, RMSE = 0.57). For a detailed description and discussion of these 3 multi-view modeling methods, please refer to Section D of Supplementary Materials.

### Analyzing toxicity associations among species by feature importance vector

The proposed framework was based on cascade forest, where each layer consisted of an ensemble of multiple forest units. The feature importance vectors extracted from the final layer represented the average of the feature importance vectors from multiple forest units in that layer. These vectors were then used to analyze the toxicity associations among different species.

**Fig. 3.**
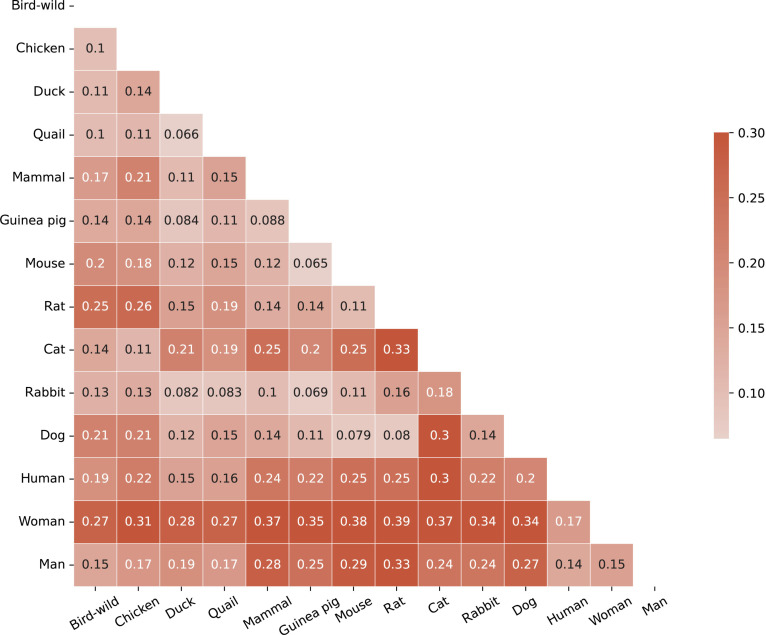
Toxicity association analysis among species under Avalon feature space.

To explore the toxicity associations among different species, we first combined the data for all toxicity endpoints by species type, treating each species as an endpoint. The proposed algorithm was then executed on each endpoint, and the feature importance vector of the model was extracted. Subsequently, pairwise Euclidean distance [25] calculations were performed on the feature importance vectors generated from all species endpoints. A similarity matrix was constructed, and a heatmap was generated (see Fig. 3). The smaller the Euclidean distance between paired feature vectors, the closer the 2 endpoints are in the feature importance distribution (FID).

The results (Fig. 3) suggest that similar species exhibit comparable toxicity associations under Avalon feature space, aligning with our understanding of species similarity. In addition, the toxicity correlations among species under other feature spaces (such as AtomPair and Morgan) also exhibit consistent similarities with Fig. 3. This indirectly validates the interpretability of the framework. For instance, in the last row of Fig. 3, the endpoints for men, women, and humans are relatively similar. Furthermore, the mouse and rat exhibit the high degree of similarity when compared to other species. Based on prior information from 59 toxicity endpoints, it appears that both humans and avian species can share oral poisoning as the administration mode, which may explain the similar FID observed between these species endpoints.

## Discussion

In this study, we proposed a novel cascade forest-based deep learning framework for modeling multi-species acute toxicity datasets of varying sizes. The framework integrated feature enhancement through data aggregation training and cascade forest layer transfer, along with sample enhancement using covariance distance measurement and a greedy neighbor search strategy. It was designed to model tabular data and balance the performance trade-off between large- and small-sized toxicity endpoints.

The experimental results indicated that the proposed method significantly outperformed all single-view comparison algorithms, achieving the 7% performance gain over the best current multi-view comparison algorithms. Friedman and Nemenyi tests were applied to validate the statistical performance differences between the proposed method and the comparison algorithms across all toxicity endpoints. Model-based ablation experiments revealed that feature enhancement was the most critical factor in improving performance. Furthermore, 3 external validation datasets (2 aquatic animals and one mammal) were employed to assess the generalization ability of the proposed method, with the best performance observed in the external mammal rat dataset. The results suggested that species consistency between the source and target domain data enhanced the model’s generalization performance in cross-species knowledge transfer.

In the multi-view data, we introduced 6 feature types and prior knowledge of each toxicity endpoint in the modeling process. The experimental results demonstrated that the proposed algorithm performed well with the Avalon, AtomPair, and Morgan feature types, whereas the integration of prior knowledge did not enhance the performance of these features. We then applied 3 multi-view modeling methods—feature concatenation, feature fusion, and the consensus framework—to model these feature types, with both the feature fusion method and consensus framework yielding further performance improvements. Finally, the feature importance distribution analysis based on species endpoint modeling revealed that human and avian species, such as birds and chickens, exhibited similar feature importance distributions, likely due to the shared administration mode or genetic similarities.

Although there is potential for further improvement in model performance, feature enhancement based on layer transfer currently imitates the concatenation method of the enhancement vector in the original cascade forest structure. This approach aims to improve the representation ability of the target domain samples by concatenating the prediction results of the source domain model with the target domain data. However, some studies have suggested that the representation ability of enhancement vectors can be further improved by employing more complex feature representation methods, such as Shapley-based feature augmentation vectors [26] or tree-based embedding vectors [27], rather than relying on a simple model output. In addition, the sample enhancement strategy, which uses covariance distance measurement and a greedy neighbor search for sample transfer at specific toxicity endpoints, overlooks relevant samples with large covariance distances. To address this, multi-task sample clustering [28,29] can be considered to implement a more accurate sample transfer for specific toxicity endpoints. These ideas will guide subsequent improvements to the model.

## Materials and Methods

### Single-view data

The multi-species acute toxicity data employed in this study were sourced from the literature published by Jain et al. [5,11]. The dataset included 80,081 unique compounds with 122,594 measurements across 59 toxicity endpoints. Table 5 provides an overview of the data, detailing the species, administration modes, toxicity types, and sample sizes. The toxicity data spanned 15 species, 8 administration modes, and 3 toxicity types.

**Table 5.** Information on the multi-species acute toxicity dataset used in this study

ID	Species	Administration modes	Toxicity type	Sample size
01	Bird-wild	Oral	LD50	338
02	Chicken	Oral	LD50	353
03	Duck	Oral	LD50	192
04	Quail	Oral	LD50	352
05	Frog	Subcutaneous	LDLo	112
**…**				
18	Mouse	Intraperitoneal	LD50	36,295
19	Mouse	Intraperitoneal	LDLo	266
20	Mouse	Intravenous	LD50	16,978
21	Mouse	Intravenous	LDLo	102
22	Mouse	Oral	LD50	23,373
**…**				
55	Dog	Oral	LD50	649
56	Dog	Oral	LDLo	187
57	Human	Oral	TDLo	140
58	Woman	Oral	TDLo	156
59	Man	Oral	TDLo	163
			Total sample size	122,594

LD50, lethal dose 50%; LDLo, lethal dose low; TDLo, toxic dose low

Species, administration modes, and toxicity types were defined as distinct toxicity endpoints, each representing a separate prediction task and corresponding to an individual dataset. The first 3 columns of the table highlight the biological significance of the dataset, and the last column indicates the sample size for each dataset. Across the 59 datasets, the total sample count was 122,594, with sample sizes varying significantly. Most datasets were small- or medium-sized, with a few being large. Therefore, the algorithm design must account for performance trade-offs across toxicity endpoints of various sizes.

Owing to space constraints, only a subset of the toxicity endpoints is shown in Table 5, with full details provided in Section E of Supplementary Materials. Additionally, Table 5 is explained in detail from a machine learning perspective in the second paragraph of the “Introduction” section, and this explanation is not repeated here. Each sample in the dataset represents the molecular expression of a compound. Because the structure of a compound can yield different expressions in various chemical spaces, multi-view data are generated.

### Multi-view data

We provided 6 commonly used molecular representations for the samples: Avalon [30], AtomPair [31], Chemopy [32], Morgan [31], ChemBERTa [33], and Unimol [34]. The first 4 were the traditional descriptors used to represent the molecular structures, whereas the latter 2 were the compound representations generated by pretrained models encoded using 1D SMILES and 3D structural information, respectively. Table 6 outlines the feature dimensions and value types for each molecular representation.

**Table 6.** Feature type, dimensions, and value types of multi-view features

Feature type	Avalon	AtomPair	Chemopy	Morgan	ChemBERTa	Unimol
Feature dimension	1,024	1,024	541	1,024	384	512
Value type	Boolean	Int	Floating	Boolean	Floating	Floating

The Avalon fingerprint [30] employed a generator to systematically enumerate specific paths and feature classes within a molecular graph. The Chemopy fingerprint [32] calculated 10 molecular descriptors to represent chemical structures, whereas AtomPairs fingerprints [31] encapsulated molecular topologies. Morgan fingerprints [31] or extended connectivity fingerprints captured the local molecular environment around each atom within a defined radius. These descriptors effectively represented diverse aspects of chemical structures and physicochemical properties, and their computation was facilitated using Python RDKit and PyChem packages.

ChemBERTa [33], based on the RoBERTa [35] transformer architecture provided by Hugging Face, was pretrained on a large dataset of 77 million chemical compounds represented in the SMILES format. Unimol [34], utilizing a 3D molecular representation learning framework within an SE(3) transformer architecture, was pretrained on a dataset comprising 209 million molecular conformations.

### Background knowledge of the proposed method

The algorithm framework proposed in this study combined multi-task learning, multi-view learning, and cascaded forest regression. To facilitate a clearer understanding of algorithm design, we first provided brief introductions to multi-task and multi-view learning and to the cascading forest regressor.

**Fig. 4.**
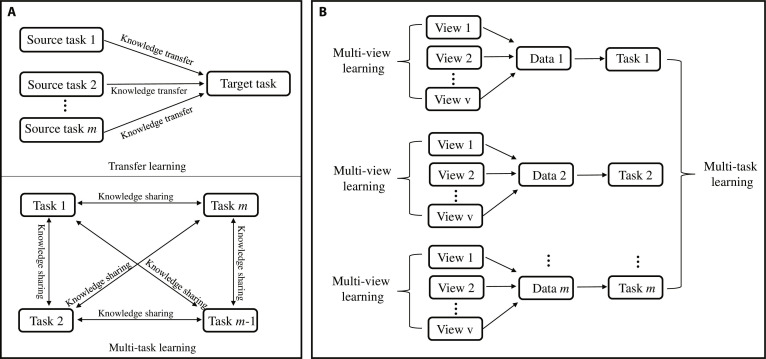
Schematic diagram of multi-task learning. (A) Differences between multi-task learning and transfer learning. (B) Combined application of multi-task learning and multi-view learning.

Multi-task learning enhances the generalization performance across multiple related tasks by leveraging shared information, enabling the development of robust, generic representations, and more powerful models for each subtask [28]. Initially, the primary motivation was to address the data sparsity in individual subtasks. Although multi-task learning shares similarities with transfer learning, key differences exist. Multi-task learning treats all subtasks equally, aiming to improve their overall performance, whereas transfer learning focuses on a single target task, using knowledge from a source task to enhance the target task’s performance. Consequently, the target task is prioritized in the transfer learning. In terms of knowledge transfer, multi-task learning facilitates knowledge sharing among all subtasks, whereas transfer learning limits knowledge transfer to interactions between the source and target tasks (Fig. 4A) [36].

Knowledge shared between tasks in multi-task learning generally takes 3 forms: features, parameters, and samples [28]. Feature-based multi-task learning identifies common features across tasks that are generic and invariant to all tasks. The parameter-based multi-task learning leveraged model parameters or prior knowledge from related tasks, such as linear model parameters, deep learning weights, or task relationships, to assist in modeling other tasks. Sample-based multi-task learning focuses on identifying related samples between tasks and improving modeling performance through data aggregation. Although most existing studies have emphasized feature- and parameter-based approaches, sample-based methods have received less attention.

Multi-view learning is often combined with multi-task learning to enhance model performance by leveraging feature insights from distinct views, where each view represents a unique feature space [37]. For example, in a compound molecular expression, a compound can exhibit different representations in various chemical spaces. Incorporating these multiple representations for the same compound provides additional information to improve the modeling performance. Figure 4B illustrates the integration of multi-view learning and multi-task learning.

Zhou and Feng [16] introduced a deep learning framework based on forest ensembles, called Deep Forest, which emulated the layer-by-layer data processing, in-model feature transformation, and model complexity of DNNs. Deep Forest comprises 2 components: multi-granularity scanning, which applies a sliding window for data augmentation, and a cascade forest, which employs a cascade structure for decision reasoning. As this study did not incorporate multi-granularity scanning, this section focuses exclusively on the cascade forest component.

**Fig. 5.**
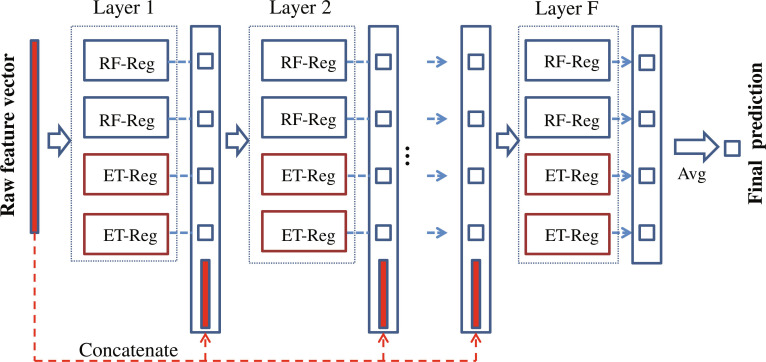
Schematic diagram of cascading forest regressor. Random forest regressor (RF-Reg), extra trees regressor (ET-Reg), and average (Avg).

This study focused on the regression task within the framework of supervised learning, as illustrated by the overall structure of the cascaded forest regressor in Fig. 5. Similar to DNNs, each layer functions as an ensemble of forest units, utilizing diverse forest algorithms to enhance the diversity of base classifiers. As shown in Fig. 5, each layer of the cascade forest predicted the input features and concatenated the output with the original feature vector to serve as the input for the next layer. This mechanism allows predictions from the previous layer to guide the training process of the subsequent layer.

The number of layers in the cascade forest was determined adaptively based on the characteristics of the data. During training, internal cross-validation was adopted to evaluate the prediction performance of each layer. If a layer outperformed the previous one, the model continued to grow; otherwise, the current layer was discarded to ensure convergence. The output layer (layer F) averaged the prediction results of all forest units in the final layer to produce the final prediction result of the cascade forest.

Compared with DNNs, cascaded forest offers several advantages, including minimal parameter tuning requirements, lower computational resource demands, enhanced model interpretability, and suitability for datasets of varying sizes. This study involved multi-task datasets with diverse sample sizes, which required careful consideration of the performance trade-offs across large, medium, and small datasets. The objective was to mitigate the overfitting risk associated with DNNs in small-sized tasks, leverage knowledge from large-sample tasks via cascaded forests to support small-sample task modeling, and thoroughly explore the interactions between critical features and their contributions to model outputs.

### Method overview

Current modeling approaches in this field rely on DNNs as the foundational framework without addressing the specific characteristics of MSATP data, leading to the following challenges. (a) The sample space of MSATP data is high-dimensional and sparse tabular data with numerous non-informative features. The rotational invariance of DNNs degrades model performance when handling such data. (b) The MSATP datasets often include a large number of small- and medium-sized toxicity endpoints, requiring careful consideration of the performance trade-offs across endpoints of varying sizes. DNNs are prone to overfitting when the data are insufficient, further complicating the modeling process.

**Fig. 6.**
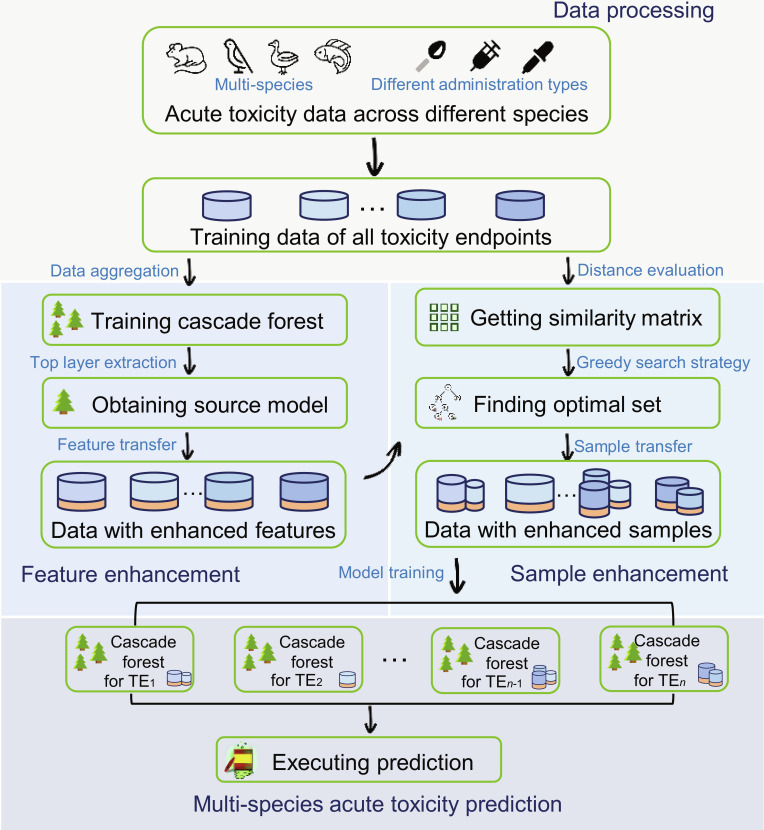
Schematic diagram of the proposed framework.

To address these challenges, this study introduced a deep learning framework based on cascaded forest (CFF-AT), specifically designed for modeling tabular data of various sizes. The framework integrates feature-, parameter-, and sample-based knowledge transfer in 4 main steps (see Fig. 6): (a) merging the training data from all toxicity endpoints to create a cascading forest enriched with knowledge; (b) extracting the first layer of the cascaded forest as the source domain model to enhance the data for each toxicity endpoint; (c) constructing a distance matrix among all toxicity endpoints using covariance measurement and employing a greedy strategy to identify the optimal neighbor set for each endpoint; and (d) using the identified neighbor sets to individually model each toxicity endpoint.

The subsequent section “Framework design” outlines the training and testing processes of the proposed framework. The section “Construction of covariance distance matrix for all toxicity endpoints” details the construction of the distance matrix among all toxicity endpoints. The section “Summary of the proposed framework” provides a summary of the proposed algorithm.

### Framework design

Algorithm 1 outlines the training process of the proposed framework. Before execution, the data for each toxicity endpoint were divided into training, validation, and testing sets at proportions of 60%, 20%, and 20%, respectively. Steps 1 to 3 merge the training and validation sets across all toxicity endpoint data as follows:

$$X=\overset{T}{\underset{i=1}{⋃}}\left(X_{{\text{train}}_{i}}+X_{{\text{valid}}_{i}}\right),$$ (3)

where *T* represents the number of all toxicity endpoints and $i\in \left[1,T\right]$ is the index for each toxicity endpoint. A cascade forest was then trained on the merged data, with its first layer extracted as the source domain model, referred to as layer 1. Steps 4 to 7 involve feature enhancement of the training and validation sets for a single toxicity endpoint using the source domain model.

$$X_{{\text{train}}_{i}}=\text{Concatenate}\left(\;X_{{\text{train}}_{i}},\;\text{Layer}1.\text{predict}\left(X_{{\text{train}}_{i}}\right)\right)$$ (4)

Equation (4) details the process of feature enhancement. The notation Layer1.predict(*) represents the prediction of the training set for the *i*th toxicity endpoint using the source domain model. In this study, the default configuration of the cascade forest regression algorithm was employed, where each layer was composed of 2 random forest units and 2 extra-tree forest units. As a result, layer 1 generated a 4D regression prediction vector for each sample. The operation *Concatenate*(*,*) combined the raw feature with the prediction vector, thereby achieving knowledge transfer from the source domain to the target domain through feature enhancement.

Steps 8 to 10 involve modeling using the training set $X_{{\text{train}}_{i}}$ of the *i*th toxicity endpoint and evaluating the model performance on the corresponding validation set $X_{{\text{valid}}_{i}}$ to establish the baseline performance of the initial modeling. Step 11 calculates the distance relationships between the *i*th toxicity endpoint and the other toxicity endpoints:

$$\text{Rank}=\text{argsort}\left(\;\text{Distance}\_\text{Matrix}\left[i\right]\;\right),$$ (5)

where $\text{Distance}\_\text{Matrix}\in R^{T*T}$, *Distance_Matrix[i]* is a row vector, and each element represents the covariance difference between the *i*th toxicity endpoint and other endpoints in the feature space. Smaller values indicate greater similarity in the feature distribution between pairwise toxicity endpoints. The argsort(*) function returns the indices of all endpoints sorted by covariance distance from the smallest to the largest.

Steps 12 to 24 describe a simple greedy algorithm aimed at iterative modeling by progressively incorporating closely related neighbor endpoint data into the current toxicity endpoint. After each iteration, the model performance was reevaluated using $X_{{\text{valid}}_{i}}$. If the performance improved, iterative training could continue; if the performance degraded, the process could be halted, and the optimal dataset (Best_train_data) for the current toxicity endpoint was finalized. In Step 25, to minimize information loss, $X_{{\text{valid}}_{i}}$ was re-included in Best_train_data, and a cascade forest was retrained for the *i*th toxicity endpoint. Upon completion of Algorithm 1, a list of models was generated with *T* cascade forests corresponding to *T* toxicity endpoints.

The prediction process of the proposed framework for all toxicity endpoints is outlined as follows. Using the list of models generated by Algorithm 1, each toxicity endpoint was predicted using its corresponding cascade forest ${\mathrm{CF}}_{i}$ in the testing set $X\_{\text{test}}_{i}$. The average modeling performance of the framework across all toxicity endpoints is expressed as follows:

$$P_{\mathrm{avg}}=\frac{1}{T}\sum_{i=1}^{T}{\mathrm{CF}}_{i}.\text{predict}\;\left(\;X_{{\text{test}}_{i}}\;\right).$$ (6)

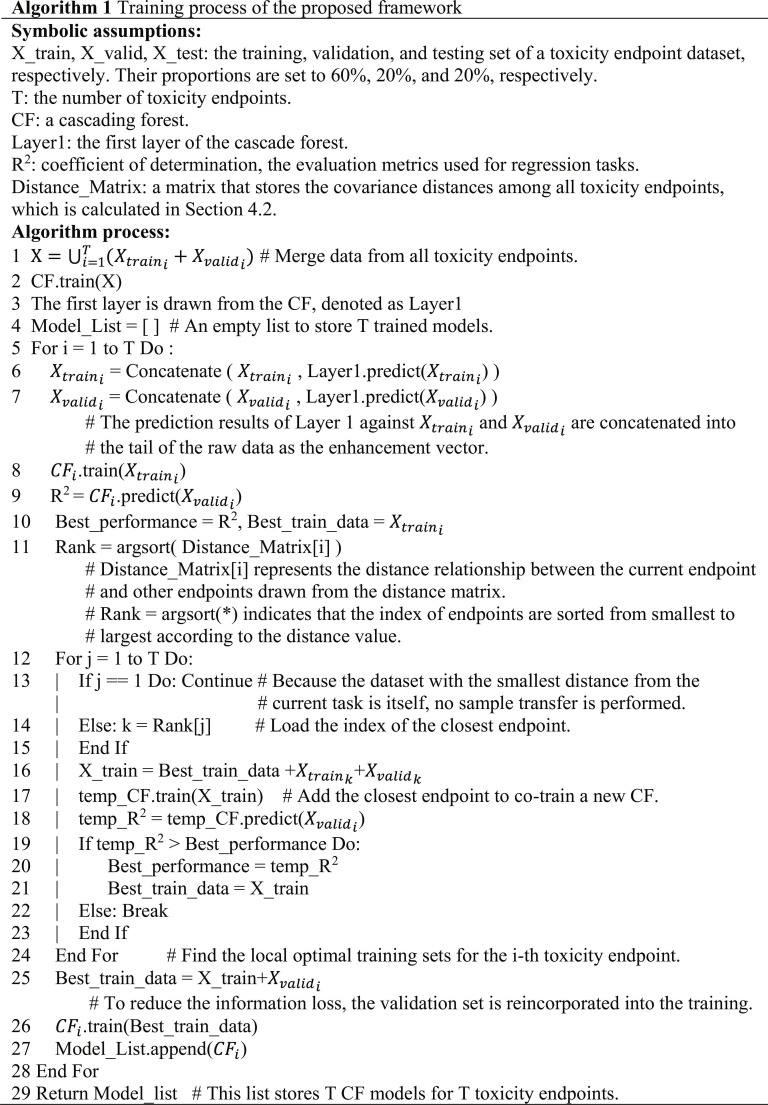


### Construction of covariance distance matrix for all toxicity endpoints

The construction of a covariance matrix for a single toxicity endpoint is described as follows. Let the feature space of a toxicity endpoint be *X*, where |*X*| represents the number of samples (rows) and *d* denotes the feature dimensions (columns). For 2 feature columns $X_{i}$ and $X_{j}$ in *X*, where *i* ≠*j* and *i*$,j\in \left[1,\;d\right]$, the covariance between them is expressed as follows:

$$\text{Covariance}\left(X_{i},\;X_{j}\right)=\frac{\sum_{n=1}^{\left|X\right|}\left(X_{i}^{n}-{\bar{X}}_{i}\right)\left(X_{j}^{n}-{\bar{X}}_{j}\right)}{\left|X\right|-1},$$ (7)

where $X_{i}^{n}$ and $X_{j}^{n}$ represent *n*th element of $X_{i}$ and $X_{j}$ (2 column vectors), and ${\bar{X}}_{i}$ and ${\bar{X}}_{j}$ represent the mean values of $X_{i}$ and $X_{j}$, respectively. Equation (7) calculates the covariance between paired feature columns. By iterating through all paired feature columns, the covariance matrix for a toxicity endpoint, denoted as *CM(X)*, can be constructed.

To construct a covariance distance matrix for all toxicity endpoints, Algorithm 2 assumes that $X^{a}\;\text{and}\;X^{b}$ are the datasets corresponding to 2 toxicity endpoints, where $a,b\in \left[1,T\right]$, and *T* is the total number of toxicity endpoints. Equation (8) calculates the mean absolute difference between the covariance matrices of $X^{a}\;\text{and}\;X^{b}$:

$$\text{Covariance Distance}\left(X^{a},\;X^{b}\right)=\text{Mean}\;\left(\left|\mathrm{CM}\left(X^{a}\right)-\mathrm{CM}\left(X^{b}\right)\right|\right).$$ (8)

where CM(*) constructs a covariance matrix for a single toxicity endpoint, |*| performs matrix subtraction and obtains the absolute difference between pairwise matrices, and Mean(*) calculates the average value of all elements in the matrix. A smaller covariance distance indicates a greater similarity in the feature distributions between the 2 toxicity endpoints. Algorithm 2 computes the covariance distances for all toxicity endpoint pairs through traversal, ultimately generating a distance matrix for all endpoints. This matrix is particularly useful when modeling small- or medium-sized toxicity endpoints, as it identifies endpoints with similar feature distributions, enabling sample transfer to enhance modeling performance.


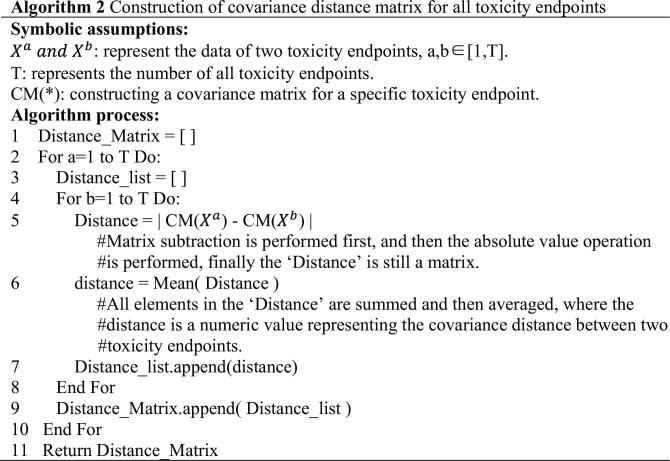


### Summary of the proposed framework

During the training process of the overall framework (Algorithm 1), merging the training sets of all toxicity endpoints serves as the source domain for knowledge transfer, with the generated source domain model encapsulating the rich knowledge. Each sample within a single toxicity endpoint acted as the target domain for knowledge acquisition, and the feature enhancement process represented feature-based knowledge transfer from the source domain to the target domain. Extraction of the covariance matrix among all toxicity endpoints enables task-relation learning, which is a parameter-based transfer method [28]. Guided by the generated covariance matrix, a greedy search strategy selected the relevant toxicity endpoint sets from the source domain data to model each endpoint individually, representing sample-based knowledge transfer. The flowchart of the proposed algorithm can be found in Section F of Supplementary Materials.

The proposed framework enhanced cascaded forest by leveraging their advantages. It retains the advantages of tree-based methods for handling small- and medium-sized tabular data while incorporating the benefits of deep learning for processing large datasets. In addition, it integrates various multi-task modeling strategies to balance the trade-off in modeling performance between small- and large-sized endpoints.

The cascade forests in the proposed framework were generated using the default configuration. Each layer contained 2 random forest regressors and 2 extra-tree regressors, with each forest unit consisting of 100 decision trees. The number of layers in the cascade forest was adaptively determined based on data characteristics. Consequently, this framework does not introduce additional hyperparameter settings.

## Data Availability

All toxicology datasets used in our study are available on the published data platform TOXRIC at https://toxric.bioinforai.tech/home. Data, code, and Supplementary Materials are also can be downloaded at https://github.com/MLDMXM2017/CFF-AT.

## Supplementary Materials

Supplementary 1 Sections A to H Tables A to H Figs. C to G
