# Supplementary material for: Multi-Task Cascade Forest Framework for Predicting Acute Toxicity across Species
Source: Research (Wash D C). 2026 Jan 15;9:1046. doi: 10.34133/research.1046 (PMC12804595; doi:10.34133/research.1046)
Supplement: Supplementary 1 — Sections A to H Tables A to H Figs. C to G [file research.1046.f1.docx]

**Multi-task Cascade Forest Framework for Predicting Acute Toxicity Across Species**

**(Supplementary Materials)**

#### Section A. Model parameter configuration

| **Table A1. Parameter configuration of all algorithms.** | | |
| --- | --- | --- |
| **Model architectures for MT-DNN** | **Parameter** | **Value** |
|  | number of hidden layers | 3 |
|  | number of neurons for each layer | 2000, 700, 500 |
|  | activation function | ReLu |
|  | optimizer | Adam |
|  | dense_size_candidates | 1500, 500, 100, 1 |
|  | learning rate (Adam) | 0.0001 |
|  | number of epochs | 50 |
|  | batch size | 128 |
| **Model architectures for DLCA** | **Parameter** | **Value** |
| Descriptor 1-3 (Fingerprints) | number of hidden layers | 4 |
|  | number of neurons for each layer | 8000, 2000, 1000, 700 |
| Descriptor 4  (RDKit physicochemical properties) | number of hidden layers | 4 |
|  | number of neurons for each layer | 8000, 2000, 1000, 700 |
|  | dropout | 0.3 |
| Descriptor 5 (SMILES) | architecture | 1D convolutional |
|  | Pooling | GlobalMaxPooling1D |
|  | number of hidden layers | 1 |
|  | number of neurons for each layer | 200 |
|  | optimizer | Adagrad |
|  | learning rate | 0.01 |
| Common configuration for above 5 networks | activation function | relu |
|  | batch size | 128 |
|  | number of epochs | 20 |
| **Model architectures for MT-GCNN** | **Parameter** | **Value** |
|  | depth | 5 |
|  | dropout | 0.3 |
|  | ffn_num_layers | 3 |
|  | hidden_size | 1700 |
| **Model architectures for Consen A** | **Parameter** | **Value** |
|  | the ensemble of MT-DNN and MT-GCNN | / |
| **Model architectures for Consen B** | **Parameter** | **Value** |
|  | the ensemble of MT-DNN, MT-GCNN, and DLCA | / |
| **Model architectures for TranAda** | **Parameter** | **Value** |
|  | estimator type | Ridge regression |
|  | estimator number | 10 |
|  | learning rate | 1 |
| **Model architectures for LinInt** | **Parameter** | **Value** |
|  | estimator type | Ridge regression |
|  | proportion | 0.5 |
| **Model architectures for CFF-AT** | **Parameter** | **Value** |
|  | base framework | Cascade forest regression |
|  | estimator number in each layer | 4 |
|  | estimator type in each layer | Random Forest*2, Extra Trees*2 |
|  | layer number | self-adaptive |
|  | task distance metric | covariance |

#### Section B. Performance comparison

| **Table B1. Performance comparison of all algorithms on 59 toxicity endpoints based on R^2^.** | | | | | | | | |
| --- | --- | --- | --- | --- | --- | --- | --- | --- |
| **Simplified Task Name** | **MT-DNN** | **MT-GCNN** | **DLCA** | **ConsenA** | **ConsenB** | **LinInt** | **TranAda** | **CFF-AT** |
| 1.bird-wild_oral_LD50 | 0.38 _(6.5)_ | 0.41 _(5.0)_ | 0.46 _(3.0)_ | 0.45 _(4.0)_ | 0.47 _(2.0)_ | 0.38 _(6.5)_ | 0.17 _(8.0)_ | 0.57 _(1.0)_ |
| 2.chicken_oral_LD50 | 0.36 _(5.0)_ | 0.31 _(8.0)_ | 0.45 _(2.0)_ | 0.41 _(4.0)_ | 0.43 _(3.0)_ | 0.33 _(6.0)_ | 0.32 _(7.0)_ | 0.56 _(1.0)_ |
| 3.duck_oral_LD50 | 0.48 _(5.0)_ | 0.44 _(6.0)_ | 0.55 _(2.0)_ | 0.52 _(4.0)_ | 0.54 _(3.0)_ | 0.33 _(8.0)_ | 0.38 _(7.0)_ | 0.68 _(1.0)_ |
| 4.quail_oral_LD50 | 0.46 _(5.0)_ | 0.44 _(6.0)_ | 0.51 _(3.0)_ | 0.49 _(4.0)_ | 0.52 _(2.0)_ | 0.35 _(8.0)_ | 0.41_(7.0)_ | 0.61 _(1.0)_ |
| 5.frog_subc_LDLo | 0.54 _(4.5)_ | 0.56 _(3.0)_ | 0.54 _(4.5)_ | 0.58 _(1.5)_ | 0.58 _(1.5)_ | 0.35 _(7.0)_ | 0.32 _(8.0)_ | 0.39 _(6.0)_ |
| 6.mammal_intrap_LD50 | 0.64 _(2.0)_ | 0.54 _(6.0)_ | 0.59 _(5.0)_ | 0.63 _(4.0)_ | 0.64 _(2.0)_ | 0.34 _(7.0)_ | 0.32 _(8.0)_ | 0.64 _(2.0)_ |
| 7.mammal_oral_LD50 | 0.63 _(2.5)_ | 0.54 _(6.0)_ | 0.57 _(5.0)_ | 0.63 _(2.5)_ | 0.64 _(1.0)_ | 0.32 _(7.0)_ | 0.28 _(8.0)_ | 0.59 _(4.0)_ |
| 8.mammal_subc_LD50 | 0.20 _(5.0)_ | 0.28 _(4.0)_ | 0.18 _(6.0)_ | 0.36 _(2.0)_ | 0.32 _(3.0)_ | 0.13 _(7.0)_ | 0.04 _(8.0)_ | 0.41 _(1.0)_ |
| 9.mammal_unrep_LD50 | 0.50 _(4.0)_ | 0.45 _(6.0)_ | 0.49 _(5.0)_ | 0.53 _(3.0)_ | 0.54 _(2.0)_ | 0.25 _(8.0)_ | 0.27 _(7.0)_ | 0.62 _(1.0)_ |
| 10.gp_intrap_LD50 | 0.57 _(3.0)_ | 0.49 _(6.0)_ | 0.56 _(4.0)_ | 0.55 _(5.0)_ | 0.58 _(2.0)_ | 0.45 _(7.0)_ | 0.40 _(8.0)_ | 0.72 _(1.0)_ |
| 11.gp_intrav_LD50 | 0.62 _(5.0)_ | 0.60 _(6.0)_ | 0.66 _(3.5)_ | 0.66 _(3.5)_ | 0.67 _(1.5)_ | 0.56 _(7.0)_ | 0.21 _(8.0)_ | 0.67 _(1.5)_ |
| 12.gp_intrav_LDLo | 0.61 _(6.0)_ | 0.57 _(7.0)_ | 0.66 _(2.5)_ | 0.62 _(4.5)_ | 0.66 _(2.5)_ | 0.62 _(4.5)_ | 0.51 _(8.0)_ | 0.75 _(1.0)_ |
| 13.gp_oral_LD50 | 0.61 _(6.0)_ | 0.63 _(5.0)_ | 0.64 _(4.0)_ | 0.67 _(2.5)_ | 0.67 _(2.5)_ | 0.45 _(8.0)_ | 0.47 _(7.0)_ | 0.76 _(1.0)_ |
| 14.gp_skin_LD50 | 0.33 _(5.0)_ | 0.32 _(6.0)_ | 0.40 _(3.0)_ | 0.37 _(4.0)_ | 0.42 _(2.0)_ | 0.21 _(8.0)_ | 0.25 _(7.0)_ | 0.48 _(1.0)_ |
| 15.gp_subc_LD50 | 0.58 _(3.5)_ | 0.48 _(6.0)_ | 0.56 _(5.0)_ | 0.58 _(3.5)_ | 0.59 _(2.0)_ | 0.41 _(8.0)_ | 0.43 _(7.0)_ | 0.67 _(1.0)_ |
| 16.gp_subc_LDLo | 0.65 _(5.5)_ | 0.68 _(4.0)_ | 0.65 _(5.5)_ | 0.70 _(2.0)_ | 0.69 _(3.0)_ | 0.56 _(8.0)_ | 0.61 _(7.0)_ | 0.81 _(1.0)_ |
| 17.mouse_intram_LD50 | 0.58 _(6.0)_ | 0.61 _(5.0)_ | 0.63 _(4.0)_ | 0.64 _(3.0)_ | 0.66 _(2.0)_ | 0.45 _(7.0)_ | 0.41 _(8.0)_ | 0.71 _(1.0)_ |
| 18.mouse_intrap_LD50 | 0.59 _(5.0)_ | 0.57 _(6.0)_ | 0.63 _(3.5)_ | 0.63 _(3.5)_ | 0.65 _(2.0)_ | 0.33 _(7.0)_ | -0.46 _(8.0)_ | 0.66 _(1.0)_ |
| 19.mouse_intrap_LDLo | 0.46 _(6.0)_ | 0.49 _(5.0)_ | 0.53 _(2.5)_ | 0.53 _(2.5)_ | 0.55 _(1.0)_ | 0.27 _(7.0)_ | 0.18 _(8.0)_ | 0.52 _(4.0)_ |
| 20.mouse_intrav_LD50 | 0.57 _(6.0)_ | 0.58 _(5.0)_ | 0.64 _(2.0)_ | 0.63 _(3.0)_ | 0.65 _(1.0)_ | 0.39 _(7.0)_ | -0.31 _(8.0)_ | 0.61 _(4.0)_ |
| 21.mouse_intrav_LDLo | 0.53 _(5.0)_ | 0.49 _(6.0)_ | 0.58 _(2.5)_ | 0.56 _(4.0)_ | 0.58 _(2.5)_ | 0.06 _(8.0)_ | 0.13 _(7.0)_ | 0.62 _(1.0)_ |
| 22.mouse_oral_LD50 | 0.51 _(5.0)_ | 0.50 _(6.0)_ | 0.55 _(4.0)_ | 0.56 _(3.0)_ | 0.58 _(2.0)_ | 0.27 _(7.0)_ | -0.53 _(8.0)_ | 0.59 _(1.0)_ |
| 23.mouse_oral_LDLo | 0.34 _(6.0)_ | 0.36 _(4.0)_ | 0.35 _(5.0)_ | 0.39 _(2.0)_ | 0.40 _(1.0)_ | 0.15 _(7.0)_ | 0.11 _(8.0)_ | 0.38 _(3.0)_ |
| 24.mouse_parent_LD50 | 0.46 _(4.0)_ | 0.44 _(5.5)_ | 0.44 _(5.5)_ | 0.53 _(1.0)_ | 0.52 _(2.0)_ | 0.33 _(8.0)_ | 0.41 _(7.0)_ | 0.51 _(3.0)_ |
| 25.mouse_skin_LD50 | 0.36 _(6.0)_ | 0.49 _(2.0)_ | 0.38 _(5.0)_ | 0.46 _(4.0)_ | 0.47 _(3.0)_ | 0.29 _(7.0)_ | 0.15 _(8.0)_ | 0.52 _(1.0)_ |
| 26.mouse_subc_LD50 | 0.59 _(5.0)_ | 0.58 _(6.0)_ | 0.62 _(4.0)_ | 0.63 _(3.0)_ | 0.65 _(2.0)_ | 0.41 _(7.0)_ | 0.14 _(8.0)_ | 0.71 _(1.0)_ |
| 27.mouse_subc_LDLo | 0.31 _(5.0)_ | 0.36 _(4.0)_ | 0.30 _(6.0)_ | 0.38 _(2.5)_ | 0.38 _(2.5)_ | 0.23 _(7.0)_ | 0.20 _(8.0)_ | 0.61 _(1.0)_ |
| 28.mouse_unrep_LD50 | 0.68 _(5.0)_ | 0.63 _(6.0)_ | 0.70 _(4.0)_ | 0.71 _(3.0)_ | 0.72 _(2.0)_ | 0.47 _(7.5)_ | 0.47 _(7.5)_ | 0.74 _(1.0)_ |
| 29.rat_intram_LD50 | 0.57 _(6.0)_ | 0.61 _(4.0)_ | 0.58 _(5.0)_ | 0.63 _(3.0)_ | 0.64 _(2.0)_ | 0.37 _(8.0)_ | 0.44 _(7.0)_ | 0.69 _(1.0)_ |
| 30.rat_intrap_LD50 | 0.56 _(5.5)_ | 0.56 _(5.5)_ | 0.59 _(4.0)_ | 0.60 _(3.0)_ | 0.62 _(2.0)_ | 0.34 _(7.0)_ | 0.04 _(8.0)_ | 0.72 _(1.0)_ |
| 31.rat_intrap_LDLo | 0.53 _(6.0)_ | 0.58 _(3.5)_ | 0.57 _(5.0)_ | 0.58 _(3.5)_ | 0.62 _(1.0)_ | 0.31 _(7.0)_ | 0.29 _(8.0)_ | 0.59 _(2.0)_ |
| 32.rat_intrav_LD50 | 0.67 _(5.0)_ | 0.65 _(6.0)_ | 0.69 _(4.0)_ | 0.70 _(3.0)_ | 0.71 _(2.0)_ | 0.44 _(7.0)_ | 0.25 _(8.0)_ | 0.77 _(1.0)_ |
| 33.rat_intrav_LDLo | 0.48 _(4.0)_ | 0.37 _(5.0)_ | 0.55 _(1.0)_ | 0.50 _(3.0)_ | 0.52 _(2.0)_ | 0.18 _(7.0)_ | 0.01 _(8.0)_ | 0.46 _(5.0)_ |
| 34.rat_oral_LD50 | 0.60 _(6.0)_ | 0.61 _(5.0)_ | 0.64 _(4.0)_ | 0.65 _(3.0)_ | 0.66 _(2.0)_ | 0.41 _(7.0)_ | 0.01 _(8.0)_ | 0.69 _(1.0)_ |
| 35.rat_oral_LDLo | 0.37 _(5.0)_ | 0.33 _(6.0)_ | 0.44 _(2.5)_ | 0.42 _(4.0)_ | 0.44 _(2.5)_ | 0.26 _(7.0)_ | 0.15 _(8.0)_ | 0.49 _(1.0)_ |
| 36.rat_skin_LD50 | 0.36 _(6.0)_ | 0.41 _(5.0)_ | 0.43 _(4.0)_ | 0.44 _(3.0)_ | 0.46 _(2.0)_ | 0.27 _(7.0)_ | -0.06 _(8.0)_ | 0.51 _(1.0)_ |
| 37.rat_subc_LD50 | 0.53 _(5.5)_ | 0.53 _(5.5)_ | 0.58 _(3.5)_ | 0.58 _(3.5)_ | 0.60 _(2.0)_ | 0.36 _(7.0)_ | 0.27 _(8.0)_ | 0.73 _(1.0)_ |
| 38.rat_subc_LDLo | 0.68 _(2.5)_ | 0.65 _(5.0)_ | 0.68 _(2.5)_ | 0.67 _(4.0)_ | 0.71 _(1.0)_ | 0.43 _(7.5)_ | 0.43 _(7.5)_ | 0.63 _(6.0)_ |
| 39.rat_unrep_LD50 | 0.56 _(6.0)_ | 0.57 _(5.0)_ | 0.60 _(4.0)_ | 0.61 _(3.0)_ | 0.62 _(2.0)_ | 0.42 _(7.5)_ | 0.42 _(7.5)_ | 0.72 _(1.0)_ |
| 40.cat_intrav_LD50 | 0.63 _(6.0)_ | 0.65 _(4.5)_ | 0.65 _(4.5)_ | 0.67 _(2.5)_ | 0.67 _(2.5)_ | 0.61 _(7.0)_ | 0.40 _(8.0)_ | 0.75 _(1.0)_ |
| 41.cat_intrav_LDLo | 0.71 _(5.0)_ | 0.66 _(7.0)_ | 0.72 _(3.0)_ | 0.72 _(3.0)_ | 0.73 _(1.0)_ | 0.67 _(6.0)_ | 0.34 _(8.0)_ | 0.72 _(3.0)_ |
| 42.cat_oral_LD50 | 0.47 _(7.0)_ | 0.46 _(8.0)_ | 0.56 _(4.5)_ | 0.52 _(6.0)_ | 0.56 _(4.5)_ | 0.62 _(2.0)_ | 0.59 _(3.0)_ | 0.71 _(1.0)_ |
| 43.cat_oral_LDLo | 0.41 _(4.5)_ | 0.39 _(6.0)_ | 0.51 _(1.0)_ | 0.45 _(3.0)_ | 0.50 _(2.0)_ | 0.21 _(8.0)_ | 0.22 _(7.0)_ | 0.41 _(4.5)_ |
| 44.rabbit_intrap_LD50 | 0.43 _(6.0)_ | 0.51 _(3.5)_ | 0.48 _(5.0)_ | 0.51 _(3.5)_ | 0.53 _(2.0)_ | 0.28 _(7.0)_ | 0.11 _(8.0)_ | 0.64 _(1.0)_ |
| 45.rabbit_intrav_LD50 | 0.72 _(4.5)_ | 0.71 _(6.0)_ | 0.72 _(4.5)_ | 0.74 _(3.0)_ | 0.75 _(2.0)_ | 0.54 _(7.0)_ | 0.45 _(8.0)_ | 0.80 _(1.0)_ |
| 46.rabbit_intrav_LDLo | 0.61 _(5.0)_ | 0.59 _(6.0)_ | 0.63 _(3.0)_ | 0.64 _(2.0)_ | 0.66 _(1.0)_ | 0.49 _(7.0)_ | 0.15 _(8.0)_ | 0.62 _(4.0)_ |
| 47.rabbit_oral_LD50 | 0.56 _(5.5)_ | 0.56 _(5.5)_ | 0.57 _(4.0)_ | 0.59 _(3.0)_ | 0.61 _(2.0)_ | 0.33 _(7.0)_ | 0.30 _(8.0)_ | 0.69 _(1.0)_ |
| 48.rabbit_oral_LDLo | 0.57 _(3.0)_ | 0.49 _(6.0)_ | 0.53 _(5.0)_ | 0.55 _(4.0)_ | 0.58 _(2.0)_ | 0.41 _(7.5)_ | 0.41 _(7.5)_ | 0.60 _(1.0)_ |
| 49.rabbit_skin_LD50 | 0.43 _(6.0)_ | 0.44 _(5.0)_ | 0.47 _(4.0)_ | 0.48 _(3.0)_ | 0.50 _(1.5)_ | 0.25 _(7.0)_ | -0.01 _(8.0)_ | 0.50 _(1.5)_ |
| 50.rabbit_skin_LDLo | 0.47 _(1.0)_ | 0.31 _(6.0)_ | 0.45 _(4.0)_ | 0.46 _(2.5)_ | 0.46 _(2.5)_ | 0.29 _(7.0)_ | 0.03 _(8.0)_ | 0.41 _(5.0)_ |
| 51.rabbit_subc_LD50 | 0.62 _(4.0)_ | 0.59 _(5.0)_ | 0.56 _(6.5)_ | 0.66 _(2.0)_ | 0.65 _(3.0)_ | 0.43 _(8.0)_ | 0.56 _(6.5)_ | 0.72 _(1.0)_ |
| 52.rabbit_subc_LDLo | 0.73 _(5.0)_ | 0.76 _(4.0)_ | 0.77 _(3.0)_ | 0.78 _(2.0)_ | 0.79 _(1.0)_ | 0.51 _(8.0)_ | 0.55 _(7.0)_ | 0.71 _(6.0)_ |
| 53.dog_intrav_LD50 | 0.64 _(6.0)_ | 0.67 _(4.0)_ | 0.66 _(5.0)_ | 0.69 _(3.0)_ | 0.70 _(2.0)_ | 0.49 _(7.0)_ | 0.20 _(8.0)_ | 0.75 _(1.0)_ |
| 54.dog_intrav_LDLo | 0.62 _(5.0)_ | 0.61 _(6.0)_ | 0.64 _(4.0)_ | 0.66 _(3.0)_ | 0.67 _(2.0)_ | 0.48 _(7.0)_ | 0.16 _(8.0)_ | 0.72 _(1.0)_ |
| 55.dog_oral_LD50 | 0.38 _(6.0)_ | 0.39 _(5.0)_ | 0.44 _(3.5)_ | 0.44 _(3.5)_ | 0.47 _(2.0)_ | 0.23 _(7.0)_ | 0.17 _(8.0)_ | 0.54 _(1.0)_ |
| 56.dog_oral_LDLo | 0.52 _(3.0)_ | 0.46 _(6.0)_ | 0.51 _(4.5)_ | 0.51 _(4.5)_ | 0.54 _(2.0)_ | 0.31 _(8.0)_ | 0.36 _(7.0)_ | 0.56 _(1.0)_ |
| 57.human_oral_TDLo | 0.32 _(1.0)_ | 0.11 _(7.0)_ | 0.27 _(5.5)_ | 0.31 _(2.0)_ | 0.3 _(3.0)_ | 0.28 _(4.0)_ | -0.34 _(8.0)_ | 0.27 _(5.5)_ |
| 58.woman_oral_TDLo | 0.20 _(5.0)_ | 0.19 _(6.0)_ | 0.13 _(7.0)_ | 0.26 _(2.5)_ | 0.26 _(2.5)_ | 0.22 _(4.0)_ | -0.64 _(8.0)_ | 0.38 _(1.0)_ |
| 59.man_oral_TDLo | 0.17 _(5.0)_ | 0.15 _(6.0)_ | 0.28 _(2.5)_ | 0.26 _(4.0)_ | 0.28 _(2.5)_ | 0.11 _(7.0)_ | -0.51 _(8.0)_ | 0.29 _(1.0)_ |
| **Average R^2^ (Rank)** | **0.51 _(4.9)_** | **0.50 _(5.4)_** | **0.54 _(4.0)_** | **0.55 _(3.2)_** | **0.57 _(2.1)_** | **0.36 _(7.0)_** | **0.20 _(7.6)_** | **0.61 _(1.9)_** |
|  |  |  |  |  |  |  |  |  |
| **Table B2. Performance comparison of all algorithms on 59 toxicity endpoints based on RMSE.** | | | | | | | | |
| **Simplified Task Name** | **MT-DNN** | **MT-GCNN** | **DLCA** | **ConsenA** | **ConsenB** | **LinInt** | **TranAda** | **CFF-AT** |
| 1.bird-wild_oral_LD50 | 0.72 _(6.0)_ | 0.71 _(5.0)_ | 0.67 _(2.5)_ | 0.68 _(4.0)_ | 0.67 _(2.5)_ | 0.76 _(7.0)_ | 0.88 _(8.0)_ | 0.64 _(1.0)_ |
| 2.chicken_oral_LD50 | 0.84 _(5.0)_ | 0.87 _(7.5)_ | 0.77 _(2.0)_ | 0.81 _(4.0)_ | 0.79 _(3.0)_ | 0.86 _(6.0)_ | 0.87 _(7.5)_ | 0.69 _(1.0)_ |
| 3.duck_oral_LD50 | 0.92 _(5.0)_ | 0.94 _(6.0)_ | 0.85 _(2.0)_ | 0.88 _(4.0)_ | 0.86 _(3.0)_ | 1.03 _(8.0)_ | 1.00 _(7.0)_ | 0.71 _(1.0)_ |
| 4.quail_oral_LD50 | 0.74 _(6.0)_ | 0.75 _(7.0)_ | 0.71 _(3.0)_ | 0.72 _(4.5)_ | 0.70 _(2.0)_ | 0.76 _(8.0)_ | 0.72 _(4.5)_ | 0.59 _(1.0)_ |
| 5.frog_subc_LDLo | 0.84 _(5.5)_ | 0.82 _(3.0)_ | 0.84 _(5.5)_ | 0.80 _(1.5)_ | 0.80 _(1.7)_ | 0.86 _(7.0)_ | 0.90 _(8.0)_ | 0.83 _(4.0)_ |
| 6.mammal_intrap_LD50 | 0.40 _(3.0)_ | 0.45 _(6.0)_ | 0.43 _(5.0)_ | 0.40 _(3.0)_ | 0.40 _(3.0)_ | 0.53 _(7.0)_ | 0.54 _(8.0)_ | 0.39 _(1.0)_ |
| 7.mammal_oral_LD50 | 0.49 _(3.0)_ | 0.54 _(6.0)_ | 0.53 _(5.0)_ | 0.48 _(1.5)_ | 0.48 _(1.5)_ | 0.66 _(7.0)_ | 0.68 _(8.0)_ | 0.51 _(4.0)_ |
| 8.mammal_subc_LD50 | 0.47 _(5.5)_ | 0.45 _(4.0)_ | 0.48 _(7.0)_ | 0.42 _(2.0)_ | 0.44 _(3.0)_ | 0.47 _(5.5)_ | 0.50 _(8.0)_ | 0.40 _(1.0)_ |
| 9.mammal_unrep_LD50 | 0.44 _(4.5)_ | 0.46 _(6.0)_ | 0.44 _(4.5)_ | 0.42 _(2.5)_ | 0.42 _(2.5)_ | 0.53 _(8.0)_ | 0.52 _(7.0)_ | 0.37 _(1.0)_ |
| 10.gp_intrap_LD50 | 0.61 _(3.0)_ | 0.66 _(6.0)_ | 0.62 _(4.0)_ | 0.63 _(5.0)_ | 0.60 _(2.0)_ | 0.71 _(7.0)_ | 0.74 _(8.0)_ | 0.51 _(1.0)_ |
| 11.gp_intrav_LD50 | 0.75 _(5.0)_ | 0.77 _(6.0)_ | 0.71 _(3.5)_ | 0.71 _(3.5)_ | 0.70 _(2.0)_ | 0.80 _(7.0)_ | 1.07 _(8.0)_ | 0.68 _(1.0)_ |
| 12.gp_intrav_LDLo | 0.65 _(5.0)_ | 0.67 _(6.0)_ | 0.61 _(2.5)_ | 0.64 _(4.0)_ | 0.61 _(2.5)_ | 0.70 _(7.0)_ | 0.79 _(8.0)_ | 0.53 _(1.0)_ |
| 13.gp_oral_LD50 | 0.72 _(6.0)_ | 0.70 _(5.0)_ | 0.69 _(4.0)_ | 0.66 _(2.5)_ | 0.66 _(2.5)_ | 0.88 _(8.0)_ | 0.87 _(7.0)_ | 0.57 _(1.0)_ |
| 14.gp_skin_LD50 | 0.77 _(5.0)_ | 0.78 _(6.0)_ | 0.74 _(3.0)_ | 0.75 _(4.0)_ | 0.72 _(2.0)_ | 0.84 _(8.0)_ | 0.83 _(7.0)_ | 0.68 _(1.0)_ |
| 15.gp_subc_LD50 | 0.81 _(4.0)_ | 0.88 _(6.5)_ | 0.83 _(5.0)_ | 0.80 _(3.0)_ | 0.79 _(2.0)_ | 0.91 _(8.0)_ | 0.88 _(6.5)_ | 0.61 _(1.0)_ |
| 16.gp_subc_LDLo | 0.75 _(5.5)_ | 0.72 _(4.0)_ | 0.75 _(5.5)_ | 0.70 _(2.5)_ | 0.70 _(2.5)_ | 0.82 _(8.0)_ | 0.77 _(7.0)_ | 0.54 _(1.0)_ |
| 17.mouse_intram_LD50 | 0.58 _(6.0)_ | 0.57 _(5.0)_ | 0.55 _(4.0)_ | 0.54 _(3.0)_ | 0.53 _(2.0)_ | 0.67 _(7.0)_ | 0.70 _(8.0)_ | 0.48 _(1.0)_ |
| 18.mouse_intrap_LD50 | 0.45 _(5.5)_ | 0.45 _(5.5)_ | 0.42 _(3.5)_ | 0.42 _(3.5)_ | 0.41 _(1.5)_ | 0.57 _(7.0)_ | 0.84 _(8.0)_ | 0.41 _(1.5)_ |
| 19.mouse_intrap_LDLo | 0.67 _(6.0)_ | 0.65 _(5.0)_ | 0.62 _(3.5)_ | 0.62 _(3.5)_ | 0.61 _(2.0)_ | 0.74 _(7.0)_ | 0.77 _(8.0)_ | 0.58 _(1.0)_ |
| 20.mouse_intrav_LD50 | 0.48 _(6.0)_ | 0.47 _(5.0)_ | 0.44 _(2.5)_ | 0.44 _(2.5)_ | 0.43 _(1.0)_ | 0.57 _(7.0)_ | 0.84 _(8.0)_ | 0.46 _(4.0)_ |
| 21.mouse_intrav_LDLo | 0.83 _(5.0)_ | 0.86 _(6.0)_ | 0.79 _(3.0)_ | 0.80 _(4.0)_ | 0.78 _(2.0)_ | 0.97 _(7.0)_ | 1.01 _(8.0)_ | 0.66 _(1.0)_ |
| 22.mouse_oral_LD50 | 0.42 _(5.0)_ | 0.43 _(6.0)_ | 0.40 _(3.5)_ | 0.40 _(3.5)_ | 0.39 _(1.5)_ | 0.52 _(7.0)_ | 0.75 _(8.0)_ | 0.39 _(1.5)_ |
| 23.mouse_oral_LDLo | 0.68 _(5.0)_ | 0.68 _(5.0)_ | 0.68 _(5.0)_ | 0.66 _(3.0)_ | 0.65 _(1.5)_ | 0.77 _(7.0)_ | 0.78 _(8.0)_ | 0.65 _(1.5)_ |
| 24.mouse_parent_LD50 | 0.71 _(5.5)_ | 0.72 _(7.5)_ | 0.72 _(7.5)_ | 0.66 _(2.0)_ | 0.67 _(3.5)_ | 0.71 _(5.5)_ | 0.67 _(3.5)_ | 0.61 _(1.0)_ |
| 25.mouse_skin_LD50 | 0.79 _(5.5)_ | 0.71 _(2.0)_ | 0.79 _(5.5)_ | 0.73 _(3.5)_ | 0.73 _(3.5)_ | 0.81 _(7.0)_ | 0.88 _(8.0)_ | 0.66 _(1.0)_ |
| 26.mouse_subc_LD50 | 0.55 _(5.0)_ | 0.56 _(6.0)_ | 0.53 _(4.0)_ | 0.52 _(3.0)_ | 0.51 _(2.0)_ | 0.67 _(7.0)_ | 0.80 _(8.0)_ | 0.47 _(1.0)_ |
| 27.mouse_subc_LDLo | 0.76 _(5.0)_ | 0.73 _(3.5)_ | 0.77 _(6.0)_ | 0.73 _(3.5)_ | 0.72 _(2.0)_ | 0.83 _(7.0)_ | 0.85 _(8.0)_ | 0.59 _(1.0)_ |
| 28.mouse_unrep_LD50 | 0.50 _(5.0)_ | 0.54 _(6.0)_ | 0.48 _(3.5)_ | 0.48 _(3.5)_ | 0.47 _(2.0)_ | 0.66 _(7.5)_ | 0.66 _(7.5)_ | 0.46 _(1.0)_ |
| 29.rat_intram_LD50 | 0.70 _(5.5)_ | 0.67 _(4.0)_ | 0.70 _(5.5)_ | 0.65 _(2.5)_ | 0.65 _(2.5)_ | 0.84 _(8.0)_ | 0.79 _(7.0)_ | 0.59 _(1.0)_ |
| 30.rat_intrap_LD50 | 0.56 _(5.5)_ | 0.56 _(5.5)_ | 0.55 _(4.0)_ | 0.54 _(3.0)_ | 0.52 _(2.0)_ | 0.69 _(7.0)_ | 0.83 _(8.0)_ | 0.45 _(1.0)_ |
| 31.rat_intrap_LDLo | 0.58 _(6.0)_ | 0.55 _(4.0)_ | 0.56 _(5.0)_ | 0.54 _(2.5)_ | 0.52 _(1.0)_ | 0.70 _(7.0)_ | 0.71 _(8.0)_ | 0.54 _(2.5)_ |
| 32.rat_intrav_LD50 | 0.56 _(5.0)_ | 0.57 _(6.0)_ | 0.54 _(3.5)_ | 0.54 _(3.5)_ | 0.52 _(2.0)_ | 0.74 _(7.0)_ | 0.85 _(8.0)_ | 0.48 _(1.0)_ |
| 33.rat_intrav_LDLo | 0.80 _(5.0)_ | 0.88 _(6.0)_ | 0.76 _(1.0)_ | 0.79 _(4.0)_ | 0.78 _(2.5)_ | 0.98 _(7.0)_ | 1.09 _(8.0)_ | 0.78 _(2.5)_ |
| 34.rat_oral_LD50 | 0.57 _(6.0)_ | 0.56 _(5.0)_ | 0.53 _(3.5)_ | 0.53 _(3.5)_ | 0.52 _(2.0)_ | 0.69 _(7.0)_ | 0.89 _(8.0)_ | 0.49 _(1.0)_ |
| 35.rat_oral_LDLo | 0.68 _(5.0)_ | 0.69 _(6.0)_ | 0.64 _(3.5)_ | 0.64 _(3.5)_ | 0.63 _(2.0)_ | 0.75 _(7.0)_ | 0.80 _(8.0)_ | 0.62 _(1.0)_ |
| 36.rat_skin_LD50 | 0.66 _(6.0)_ | 0.63 _(5.0)_ | 0.62 _(3.5)_ | 0.62 _(3.5)_ | 0.61 _(2.0)_ | 0.67 _(7.0)_ | 0.80 _(8.0)_ | 0.56 _(1.0)_ |
| 37.rat_subc_LD50 | 0.68 _(5.5)_ | 0.68 _(5.5)_ | 0.65 _(4.0)_ | 0.64 _(3.0)_ | 0.63 _(2.0)_ | 0.81 _(7.0)_ | 0.86 _(8.0)_ | 0.52 _(1.0)_ |
| 38.rat_subc_LDLo | 0.66 _(3.0)_ | 0.69 _(6.0)_ | 0.66 _(3.0)_ | 0.67 _(5.0)_ | 0.63 _(1.0)_ | 0.82 _(7.0)_ | 0.83 _(8.0)_ | 0.66 _(3.0)_ |
| 39.rat_unrep_LD50 | 0.64 _(6.0)_ | 0.62 _(5.0)_ | 0.60 _(4.0)_ | 0.59 _(3.0)_ | 0.58 _(2.0)_ | 0.74 _(7.5)_ | 0.74 _(7.5)_ | 0.51 _(1.0)_ |
| 40.cat_intrav_LD50 | 0.80 _(6.0)_ | 0.78 _(4.5)_ | 0.78 _(4.5)_ | 0.76 _(3.0)_ | 0.75 _(2.0)_ | 0.85 _(7.0)_ | 1.04 _(8.0)_ | 0.68 _(1.0)_ |
| 41.cat_intrav_LDLo | 0.68 _(4.0)_ | 0.74 _(6.0)_ | 0.66 _(2.0)_ | 0.67 _(3.0)_ | 0.65 _(1.0)_ | 0.76 _(7.0)_ | 1.08 _(8.0)_ | 0.70 _(5.0)_ |
| 42.cat_oral_LD50 | 0.83 _(7.0)_ | 0.84 _(8.0)_ | 0.76 _(2.5)_ | 0.79 _(5.0)_ | 0.76 _(2.5)_ | 0.77 _(4.0)_ | 0.80 _(6.0)_ | 0.68 _(1.0)_ |
| 43.cat_oral_LDLo | 0.69 _(5.0)_ | 0.70 _(6.0)_ | 0.64 _(1.5)_ | 0.67 _(4.0)_ | 0.64 _(1.5)_ | 0.75 _(7.0)_ | 0.77 _(8.0)_ | 0.66 _(3.0)_ |
| 44.rabbit_intrap_LD50 | 0.66 _(6.0)_ | 0.62 _(4.0)_ | 0.63 _(5.0)_ | 0.61 _(3.0)_ | 0.60 _(2.0)_ | 0.75 _(7.0)_ | 0.83 _(8.0)_ | 0.51 _(1.0)_ |
| 45.rabbit_intrav_LD50 | 0.63 _(5.0)_ | 0.64 _(6.0)_ | 0.62 _(4.0)_ | 0.61 _(3.0)_ | 0.59 _(2.0)_ | 0.81 _(7.0)_ | 0.89 _(8.0)_ | 0.53 _(1.0)_ |
| 46.rabbit_intrav_LDLo | 0.74 _(4.5)_ | 0.75 _(6.0)_ | 0.71 _(2.5)_ | 0.71 _(2.5)_ | 0.69 _(1.0)_ | 0.83 _(7.0)_ | 1.09 _(8.0)_ | 0.74 _(4.5)_ |
| 47.rabbit_oral_LD50 | 0.62 _(6.0)_ | 0.61 _(5.0)_ | 0.60 _(3.5)_ | 0.60 _(3.5)_ | 0.58 _(2.0)_ | 0.74 _(7.0)_ | 0.76 _(8.0)_ | 0.50 _(1.0)_ |
| 48.rabbit_oral_LDLo | 0.58 _(2.5)_ | 0.63 _(6.0)_ | 0.61 _(5.0)_ | 0.59 _(4.0)_ | 0.58 _(2.5)_ | 0.68 _(7.5)_ | 0.68 _(7.5)_ | 0.55 _(1.0)_ |
| 49.rabbit_skin_LD50 | 0.56 _(6.0)_ | 0.55 _(5.0)_ | 0.54 _(4.0)_ | 0.53 _(2.5)_ | 0.53 _(2.5)_ | 0.64 _(7.0)_ | 0.73 _(8.0)_ | 0.52 _(1.0)_ |
| 50.rabbit_skin_LDLo | 0.67 _(1.0)_ | 0.77 _(7.0)_ | 0.70 _(5.0)_ | 0.68 _(3.0)_ | 0.68 _(3.0)_ | 0.74 _(6.0)_ | 0.86 _(8.0)_ | 0.68 _(3.0)_ |
| 51.rabbit_subc_LD50 | 0.81 _(4.0)_ | 0.83 _(5.0)_ | 0.86 _(6.0)_ | 0.76 _(2.0)_ | 0.78 _(3.0)_ | 1.00 _(8.0)_ | 0.88 _(7.0)_ | 0.69 _(1.0)_ |
| 52.rabbit_subc_LDLo | 0.66 _(6.0)_ | 0.61 _(4.5)_ | 0.61 _(4.5)_ | 0.59 _(2.0)_ | 0.57 _(1.0)_ | 0.80 _(8.0)_ | 0.77 _(7.0)_ | 0.60 _(3.0)_ |
| 53.dog_intrav_LD50 | 0.66 _(6.0)_ | 0.64 _(4.5)_ | 0.64 _(4.5)_ | 0.62 _(2.0)_ | 0.60 _(3.0)_ | 0.79 _(7.0)_ | 0.98 _(8.0)_ | 0.54 _(1.0)_ |
| 54.dog_intrav_LDLo | 0.71 _(5.0)_ | 0.72 _(6.0)_ | 0.70 _(4.0)_ | 0.68 _(3.0)_ | 0.67 _(2.0)_ | 0.86 _(7.0)_ | 1.10 _(8.0)_ | 0.63 _(1.0)_ |
| 55.dog_oral_LD50 | 0.77 _(6.0)_ | 0.76 _(5.0)_ | 0.73 _(3.5)_ | 0.73 _(3.5)_ | 0.71 _(2.0)_ | 0.86 _(7.0)_ | 0.89 _(8.0)_ | 0.66 _(1.0)_ |
| 56.dog_oral_LDLo | 0.83 _(3.0)_ | 0.88 _(6.0)_ | 0.84 _(4.5)_ | 0.84 _(4.5)_ | 0.82 _(2.0)_ | 0.95 _(8.0)_ | 0.91 _(7.0)_ | 0.76 _(1.0)_ |
| 57.human_oral_TDLo | 1.27 _(1.0)_ | 1.44 _(7.0)_ | 1.32 _(4.0)_ | 1.29 _(2.5)_ | 1.29 _(2.5)_ | 1.33 _(5.0)_ | 1.81 _(8.0)_ | 1.34 _(6.0)_ |
| 58.woman_oral_TDLo | 1.04 _(4.0)_ | 1.05 _(5.0)_ | 1.09 _(6.0)_ | 1.01 _(1.5)_ | 1.01 _(1.5)_ | 1.17 _(7.0)_ | 1.68 _(8.0)_ | 1.03 _(3.0)_ |
| 59.man_oral_TDLo | 1.11 _(5.0)_ | 1.13 _(6.0)_ | 1.04 _(1.5)_ | 1.06 _(3.5)_ | 1.04 _(1.5)_ | 1.20 _(7.0)_ | 1.57 _(8.0)_ | 1.06 _(3.5)_ |
| **Average RMSE (Rank)** | **0.69 _(5.0)_** | **0.70 _(5.5)_** | **0.67 _(4.0)_** | **0.66 _(3.2)_** | **0.65 _(2.1)_** | **0.78 _(7.0)_** | **0.87 _(7.6)_** | **0.61 _(1.7)_** |

Tables B1 and B2 present the R^2^ and RMSE values for all algorithms across the 59 toxicity endpoints. Each row represents the model performance for a specific endpoint, with values in parentheses indicating the performance rank. A rank of 1 denotes the best performance, 2 denotes the second-best, and so forth. If multiple algorithms achieve equal performance, an average rank is assigned. The last row of each table summarizes the average performance and rank of all models across the 59 toxicity endpoints.

#### Section C. Friedman test and Nemenyi test for Tables B1 and B2

To further validate the effectiveness of the proposed method, the Friedman test and Nemenyi test were conducted to assess the performance differences between the proposed method and comparison algorithms. The Friedman test, serving as a non-parametric statistical test, evaluated whether significant differences were observed in the prediction performance of multiple models across multiple datasets by analyzing rank data.

Consider N datasets and K algorithms, where $i\in\left[ 1,N \right] \mathrm{and} j\in\left[ 1,K \right]$, $R_{j}^{i}$ denotes the rank of the *j-th* algorithm on the *i-th* dataset, and $R_{j}$ represents the average rank of the *j-th* algorithm across all datasets (Equation (1)). The initial calculation for the Friedman test is described by Equation (2), whereas Iman et al. considered this formula to be overly conservative and proposed an improved version, represented by Equation (3).

$R_{j}=\frac{1}{N}\sum_{i=1}^{N} R_{j}^{i}, \forall i\in\left[ 1,N \right] and j\in\left[ 1,K \right]$ (1)

$X_{F}^{2}=\frac{12N}{K(K+1)} \left[ \sum_{j} R_{j}^{2}-\frac{K{(K+1)}^{2}}{4} \right]$ (2)

$F_{F}=\frac{(N-1)X_{F}^{2}}{N\left( K-1 \right)-X_{F}^{2}}$ (3)

Using Table B1 of Section B as an example, with K=8 and N=59, the average rank of each algorithm is provided in the last row of Table B1. The Friedman test calculations for Table B1 are presented in Equations (4) and (5).

$X_{F}^{2}=\frac{12*59}{8(8+1)}*\left( {4.9}^{2}+{5.4}^{2}+{4.0}^{2}+{3.2}^{2}+{2.1}^{2}+{7.0}^{2}+{7.6}^{2}+{1.9}^{2}-\frac{8*\left( 8+1 \right)^{2}}{4} \right)=316.53$ (4)

$F_{F}=\frac{(59-1)X_{F}^{2}}{59\left( 8-1 \right)-X_{F}^{2}}=190.31$ (5)

Using the degrees of freedom (K-1) and (K-1)*(N-1) in the F distribution, the threshold value F(7,406) at α=0.05 was 2.03, as found in the standard statistical tables. Under the null hypothesis, the Friedman test assumes that all algorithms perform equally. However, the statistical result from Equation (5) exceeded the threshold value of 2.03, allowing us to reject the null hypothesis and conclude that there were significant differences in R^2^ performance across all models for the 59 toxicity endpoints at a 95% confidence level. Additionally, the Friedman test was performed for Table B2 of Section B, demonstrating significant differences in the RMSE performance among all models. The calculation process was not repeated here.

When the null hypothesis of the Friedman test was rejected, a follow-up Nemenyi test was conducted to analyze the performance differences between pairwise algorithms. A significant statistical difference exists if the difference in their average ranks exceeds the Critical Difference (CD) calculated using Equation (6). Referring to Table B1, CD at $q_{\alpha}=0.05$ was computed as $CD=3.031*\sqrt{\frac{8*\left( 8+1 \right)}{6*59}}=3.0$. Using the last row of Table B1 as an example, the rank difference between the proposed algorithm CFF-AT and the comparison algorithm TranAda was $\left| 1.9-7.6 \right|=5.7>3.0$, indicating a significant performance difference between these two methods in terms of R^2^.

$CD=q_{\alpha}\sqrt{\frac{K(K+1)}{6N}}$ (6)

| **Table C1. Critical values for the two-tailed Nemenyi test.** | | | | | | | | | |
| --- | --- | --- | --- | --- | --- | --- | --- | --- | --- |
| Number of algorithms | 2 | 3 | 4 | 5 | 6 | 7 | **8** | 9 | 10 |
| $q_{0.05}$ | 1.960 | 2.343 | 2.569 | 2.728 | 2.850 | 2.949 | **3.031** | 3.102 | 3.164 |
| $q_{0.1}$ | 1.645 | 2.052 | 2.291 | 2.459 | 2.589 | 2.693 | 2.780 | 2.855 | 2.920 |


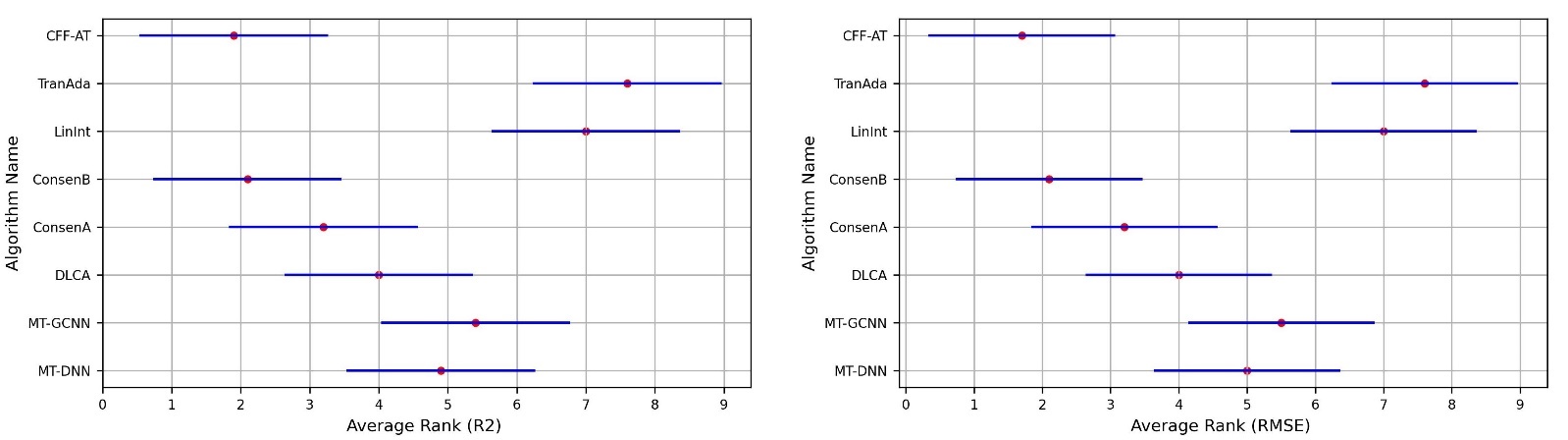


**Fig. C1** Performance rank difference between pairwise algorithms under R^2^ and RMSE.

Figure C1 illustrates the performance rank differences among all algorithms for clarity. The Y-axis represents the algorithm names, and the X-axis shows the rank values, where smaller values indicate better performance. The red dots correspond to the rank values of each algorithm, and the length of each line is twice that of CD. A significant performance difference was observed when the horizontal distance between the two red dots exceeded half of the line length. As shown in Figure C1, the performance of most of the comparison algorithms lagged significantly behind, except for ConsenB, whose average performance was relatively close to that of CFF-AT.

#### Section D. Exploration of multi-view data modeling


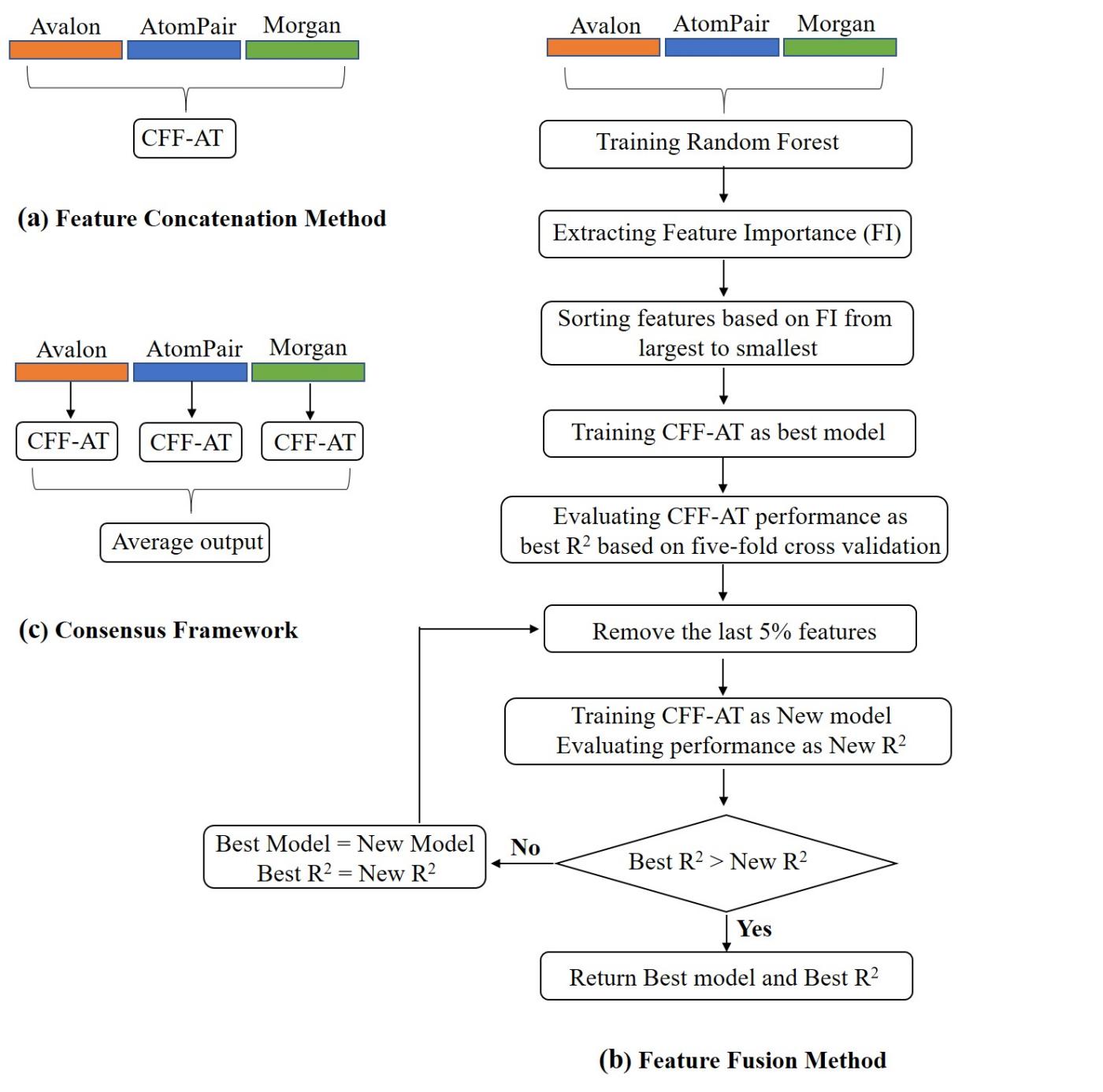


**Fig. D1** Overview of multi-view data modeling approach.

This experiment explored the modeling performance of CFF-AT with multi-view data using three methods, as illustrated in Figure D1. Method (a), Feature Concatenation [5], concatenates the representations of all samples in Avalon, AtomPair, and Morgan feature spaces to train a single model. Method (b), Feature Fusion, extends method (a) by first concatenating the three feature types to train a random forest, extracting the feature importance values (FIV), and reordering features based on FIV. It then iteratively removed the bottom 5% of the features with the lowest ranks and tested for performance improvement until convergence. Method (b) [6] effectively combines iterative feature dimensionality reduction with CFF-AT. Method (c), Consensus Framework [7], trains separate models for each of the three feature types and averages their outputs within the overall framework.

Table D1 compares the performance of the three multi-view modeling methods. Model 1 represents the performance of CFF-AT using a single view (Avalon) as a benchmark. Model 2 corresponds to the Feature Concatenation method, where the performance slightly decreases, likely owing to the feature redundancy caused by concatenation. Model 3, corresponding to the Feature Fusion method, improves the performance by iteratively eliminating redundant features, ultimately using only 10% of the fused features. These highlights significant information redundancy in the feature spaces of Avalon, AtomPair, and Morgan. Model 4 represents the consensus framework, which achieved the best performance by averaging the outputs of the three CFF-AT models trained on each feature type.

| **Table D1.** Performance comparison of three multi-view modeling methods. | | | | | |
| --- | --- | --- | --- | --- | --- |
| ID | Model Name | Input Feature Type | Num of features used | R2 | RMSE |
| 1 | CFF-AT | Avalon | 1024 | 0.61 | 0.61 |
| 2 | CFF-AT-Concatenation | Avalon, AtomPair, Morgan | 1024*3=3072 | 0.60 | 0.61 |
| 3 | CFF-AT-Fusion | Avalon, AtomPair, Morgan | 1024*3*0.1=307 | 0.62 | 0.60 |
| 4 | CFF-AT-Consensus | Avalon, AtomPair, Morgan | 1024*3=3072 | **0.64** | **0.57** |

#### Section E. Information of multi-species acute toxicity dataset

| **Table E1 Information on the multi-species acute toxicity dataset** | | | | |
| --- | --- | --- | --- | --- |
| ID | Species | Administration | Toxicity Type | Size |
| 01 | bird-wild | oral | LD50 | 338 |
| 02 | chicken | oral | LD50 | 353 |
| 03 | duck | oral | LD50 | 192 |
| 04 | quail | oral | LD50 | 352 |
| 05 | frog | subcutaneous | LDLo | 112 |
| 06 | mammal (species unspecified) | intraperitoneal | LD50 | 545 |
| 07 | mammal (species unspecified) | oral | LD50 | 674 |
| 08 | mammal (species unspecified) | subcutaneous | LD50 | 125 |
| 09 | mammal (species unspecified) | unreported | LD50 | 1129 |
| 10 | guinea pig | intraperitoneal | LD50 | 248 |
| 11 | guinea pig | intravenous | LD50 | 153 |
| 12 | guinea pig | intravenous | LDLo | 121 |
| 13 | guinea pig | oral | LD50 | 793 |
| 14 | guinea pig | skin | LD50 | 176 |
| 15 | guinea pig | subcutaneous | LD50 | 169 |
| 16 | guinea pig | subcutaneous | LDLo | 179 |
| 17 | mouse | intramuscular | LD50 | 571 |
| 18 | mouse | intraperitoneal | LD50 | 36295 |
| 19 | mouse | intraperitoneal | LDLo | 266 |
| 20 | mouse | intravenous | LD50 | 16978 |
| 21 | mouse | intravenous | LDLo | 102 |
| 22 | mouse | oral | LD50 | 23373 |
| 23 | mouse | oral | LDLo | 264 |
| 24 | mouse | parenteral | LD50 | 302 |
| 25 | mouse | skin | LD50 | 214 |
| 26 | mouse | subcutaneous | LD50 | 6769 |
| 27 | mouse | subcutaneous | LDLo | 252 |
| 28 | mouse | unreported | LD50 | 1739 |
| 29 | rat | intramuscular | LD50 | 300 |
| 30 | rat | intraperitoneal | LD50 | 5021 |
| 31 | rat | intraperitoneal | LDLo | 318 |
| 32 | rat | intravenous | LD50 | 2472 |
| 33 | rat | intravenous | LDLo | 135 |
| 34 | rat | oral | LD50 | 10190 |
| 35 | rat | oral | LDLo | 322 |
| 36 | rat | skin | LD50 | 835 |
| 37 | rat | subcutaneous | LD50 | 1896 |
| 38 | rat | subcutaneous | LDLo | 174 |
| 39 | rat | unreported | LD50 | 806 |
| 40 | cat | intravenous | LD50 | 261 |
| 41 | cat | intravenous | LDLo | 159 |
| 42 | cat | oral | LD50 | 171 |
| 43 | cat | oral | LDLo | 142 |
| 44 | rabbit | intraperitoneal | LD50 | 131 |
| 45 | rabbit | intravenous | LD50 | 792 |
| 46 | rabbit | intravenous | LDLo | 346 |
| 47 | rabbit | oral | LD50 | 894 |
| 48 | rabbit | oral | LDLo | 249 |
| 49 | rabbit | skin | LD50 | 1495 |
| 50 | rabbit | skin | LDLo | 181 |
| 51 | rabbit | subcutaneous | LD50 | 156 |
| 52 | rabbit | subcutaneous | LDLo | 241 |
| 53 | dog | intravenous | LD50 | 468 |
| 54 | dog | intravenous | LDLo | 360 |
| 55 | dog | oral | LD50 | 649 |
| 56 | dog | oral | LDLo | 187 |
| 57 | human | oral | TDLo | 140 |
| 58 | woman | oral | TDLo | 156 |
| 59 | man | oral | TDLo | 163 |
| **Total sample size** | | | | **122594** |

**Note:** Lethal Dose Low (LDLo), Lethal Dose 50 (LD50), Toxic Does Low (TDLo).

1. **Data Preprocessing**

The multi-species acute toxicity data involved in this study were derived from the published literature. Data preprocessing involved data extraction, unit standardization, and compound screening.

Firstly, Jain et al. extracted the acute toxicity measurements from the publicly available ChemIDplus database, which is a key chemical database under the Toxicology Data Network (TOXNET) of the United States National Library of Medicine. ChemIDplus consolidates acute toxicity data and related compound information from multiple sources, including median lethal dose (LD50), lethal dose low (LDLo), and toxic dose low (TDLo).

Secondly, various types of measurements were standardized to units of mg/kg, ug/kg, or ng/kg, and all values were ultimately converted to mg/kg. To mitigate the magnitude differences, the toxicity values were further converted into dimensionless values of −log(mol/kg). This logarithmic transformation not only compresses the dynamic range of the data but also effectively reduces the impact of outliers on statistical analysis through molar concentration normalization.

Thirdly, compounds in the dataset were filtered and standardized. This process involved removing salts, solvents, counterions, large organic compounds (molecular weight ≥ 2000 Da), mixtures, and inorganic compounds, as well as standardizing specific chemical structures (such as aromatic rings, nitro groups, etc.).

To ensure data quality, toxicity endpoints with fewer than 100 measurements were excluded, resulting in a final dataset containing 80,081 unique compounds and 122,594 measurements across 59 toxicity endpoints. Table E1 provides an overview of the data, detailing the species, administration modes, toxicity types, and sample sizes.

1. **Analysis of Missing Values and Outliers**

The feature space of the dataset expressed the chemical structure of compounds, and the label space expressed the toxicity values of these compounds at different toxicity endpoints. The dataset containing 80,081 unique compounds and 122,594 measurements across 59 toxicity endpoints, where one measurement corresponds to one sample.

The feature representation of the compounds is calculated using specific methods without missing values. However, not all compounds can be measured at 59 toxicity endpoints. In other words, if complete measurements are conducted, the total number of samples for 59 toxicity endpoints should be 80,081*59. But for some toxicity endpoints, it is impossible to measure the toxicity of a large number of compounds, such as human oral toxicity (endpoints 57-59 of Table E1). Therefore, the toxicity values (label) in the dataset exhibit a high degree of sparsity with a substantial number of missing entries. More specifically, there are significant differences in the number of measurements for each toxicity endpoint. To handle the aforementioned situation, we employ multi-task learning to model each endpoint individually, which helps to alleviate the adverse effects caused by label sparsity during the modeling process.

We visualize the distribution of the labels. The left histogram in Fig.E1 shows that the trend of label values follows a normal distribution. The right box plot in Fig.E1 demonstrates that there are a few outliers beyond the upper and lower bounds of the interquartile range. However, we believe that a small number of outliers caused by non-measurement errors in large datasets should be retained to restore the authenticity of the data.


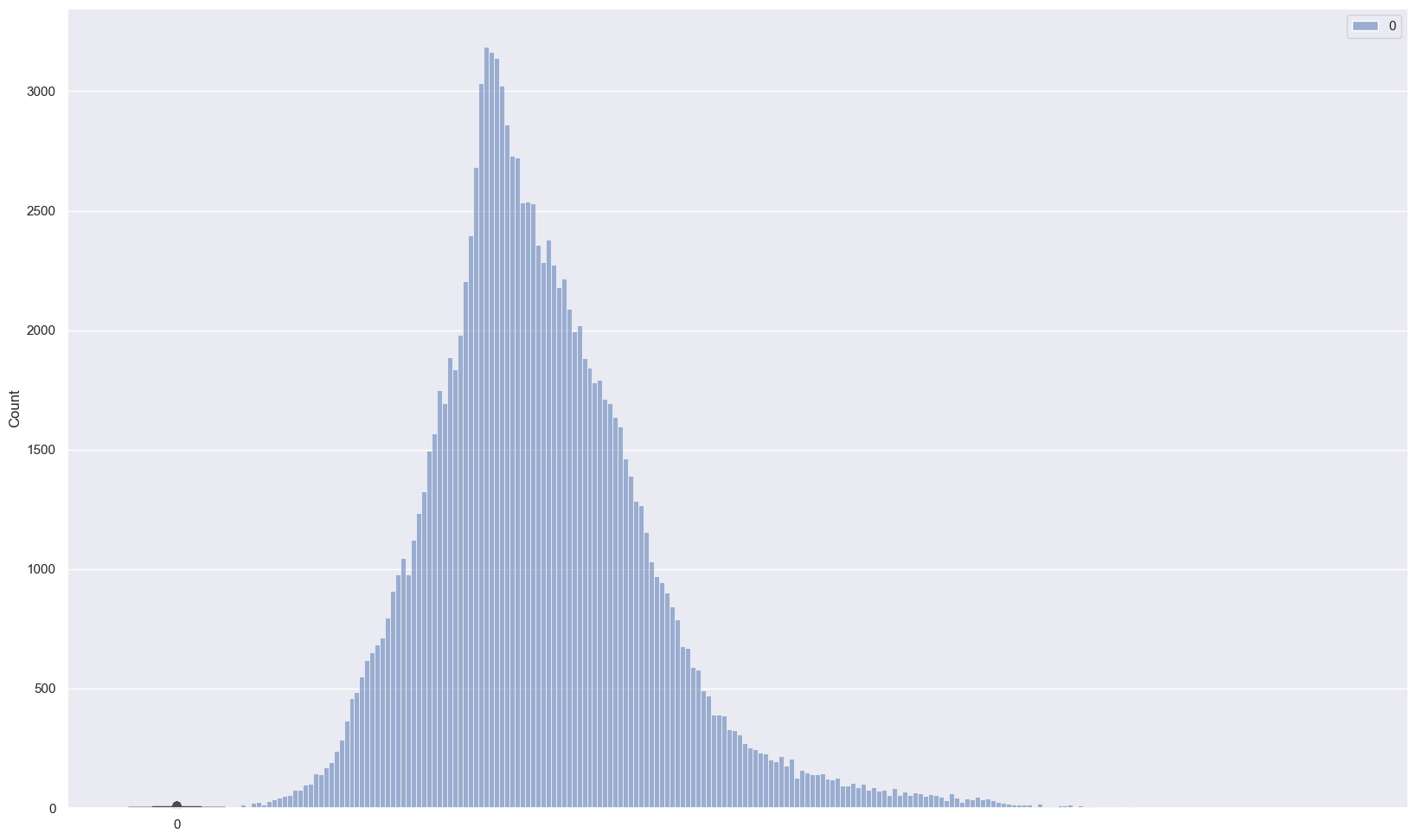

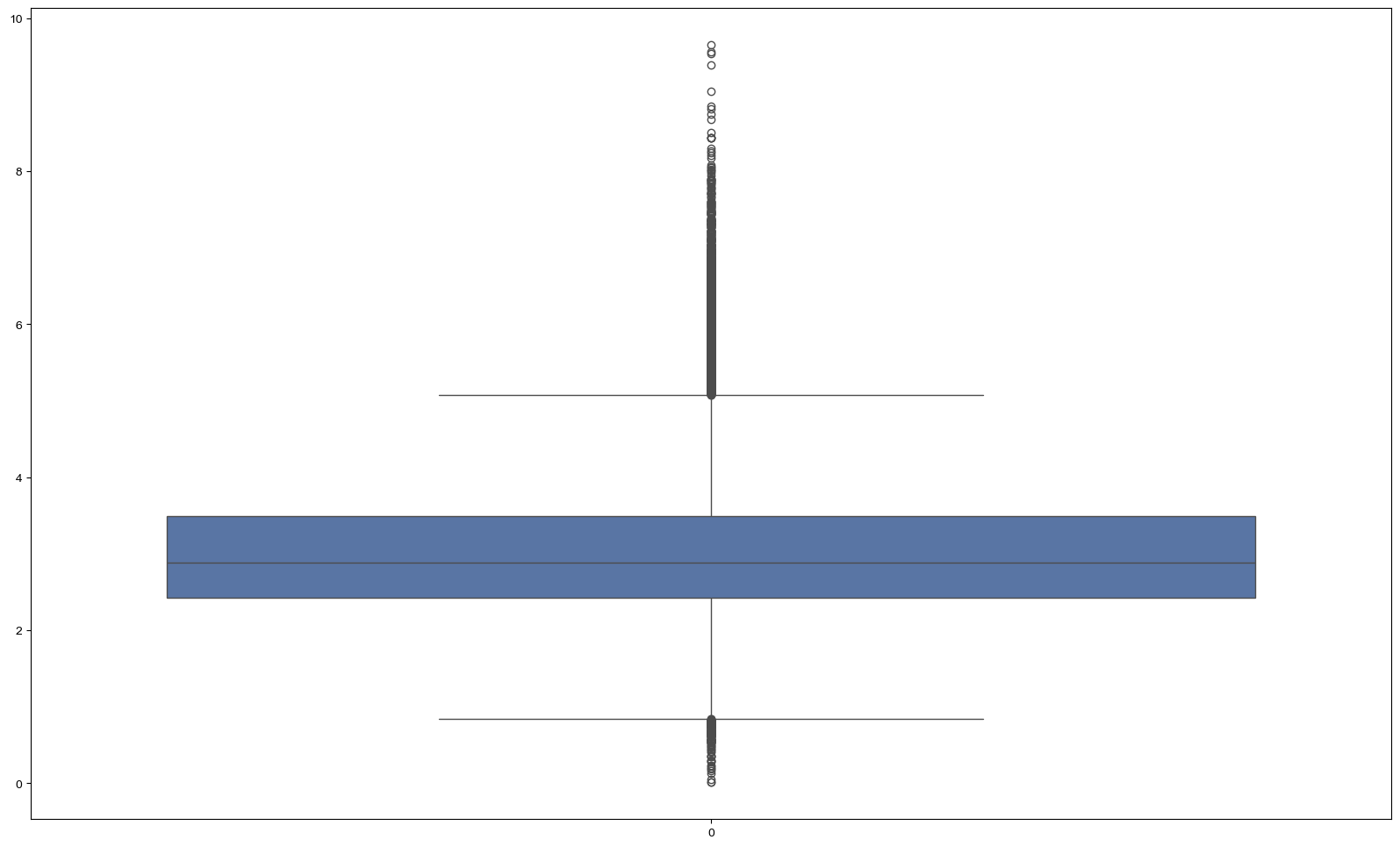


Fig.E1. The distribution of label values.

#### Section F. Algorithm flowchart


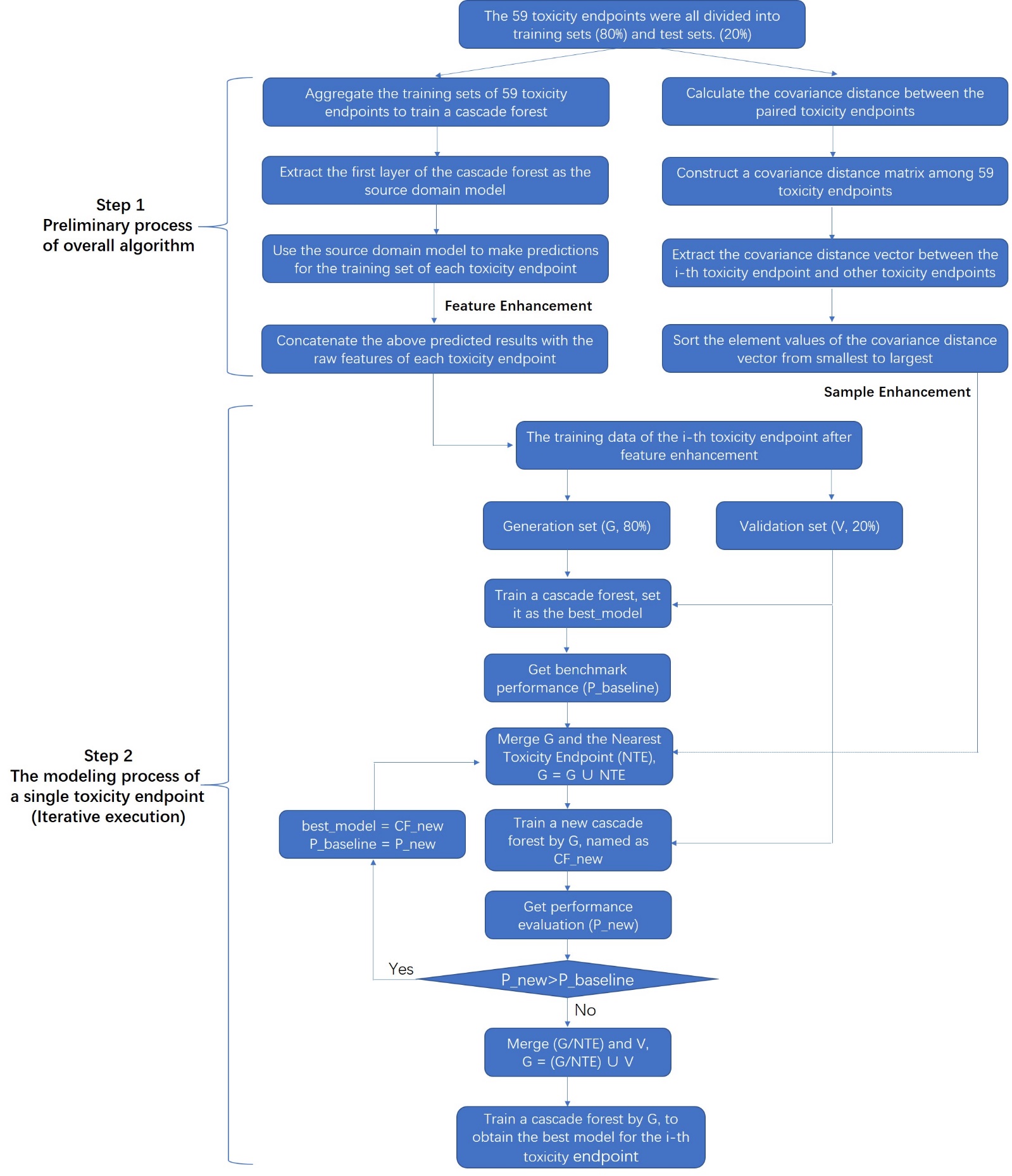


Fig.F1. The flowchart of the proposed algorithm.

#### Section G. Supplementary experiments during the second revision process

1. **Scaffold-CV**

Compared with 5-fold cross-validation (R^2^ = 0.61, RMSE = 0.61), when using Scaffold-CV with reserving 20% of the samples as the test set, the model performance does significantly decline (R^2^ = 0.42, RMSE = 0.75). The main reason is that cross-validation divides the training set and testing set based on the data distribution, allowing the model to make effective predictions. However, Scaffold-CV divides based on the molecular skeleton, and its data partitioning strategy is stricter. The distribution pattern of the testing set may rarely be reflected in the training set, deteriorating model performance. Additionally, all the published literatures based on this dataset just report results using cross-validation methods. To enable effective comparison with the comparison algorithms, we mainly report the results of cross-validation in the manuscript.

1. **Y-randomization experiments**

We conducted 30 runs of Y-randomization experiments on the proposed method. The steps are as follows:

(a) Under the Avalon feature space, set the original modeling result of the proposed method as the benchmark (R^2^ = 0.61).

(b) Keep the feature matrix X unchanged and randomly shuffle the element values of the Y matrix for each toxicity endpoint.

(c) Use the proposed method to model the new dataset and perform 5-fold cross-validation to obtain the new performance.

(d) Repeat steps (b) and (c), and check the number of times that the new performance exceeds the benchmark performance after conducting 30 runs of Y-randomization experiments.

After the above experiments, no performance of Y-randomization experiments exceeded the benchmark performance. Therefore, it can be considered that the p-value corresponding to the experiment (0/30) is much smaller than 0.05, proving that the proposed method has truly discovered the mapping relationship between the compounds and the toxicity values.

1. **Applicability domain analysis**

The reliability of a model’s prediction depends on whether the queried compound falls within the Applicability Domain (AD) defined by the model. Following the published literatures, we quantify the AD of our model using Tanimoto similarity based on Avalon molecular fingerprint. The AD threshold $S_{T}$ is defined as the range from 0.1 to 0.7 for evaluation. For each test compound, we identify its *k* nearest neighbors (*k*=10) in the training set based on Tanimoto similarity scores. The test compound is classified as in-AD if the average similarity of these top *k* neighbors exceeds the calculated threshold $S_{T}$.

The AD of the model was assessed to identify the chemical space where it achieves reliable predictions. By evaluating a range of AD thresholds $S_{T}$ from 0.1 to 0.7, we analyzed the corresponding variations in predictive performance and the coverage rates of compounds within the AD in some representative endpoints of each specie (Fig. G1 and G2). As the threshold $S_{T}$ increases, the coverage of molecules in the AD decreases sharply, while prediction accuracy improves. For instance, in Dataset2 (chicken-oral-LD50, the second item of Fig.G1 and G2), when the threshold $S_{T}$ increases, R2 rises from 0.57 to 0.73 and RMSE decreases from 0.69 to 0.38. This demonstrates that stricter AD thresholds effectively screen high-confidence predictions by narrowing the chemical space. By balancing prediction accuracy and coverage, *S_T_*=0.5 was selected as the globally optimal parameter, achieving 39.44% in-AD coverage while maintaining strong predictive performance, thus ensuring both model reliability and practical utility. A similar trend was observed across the other endpoints.


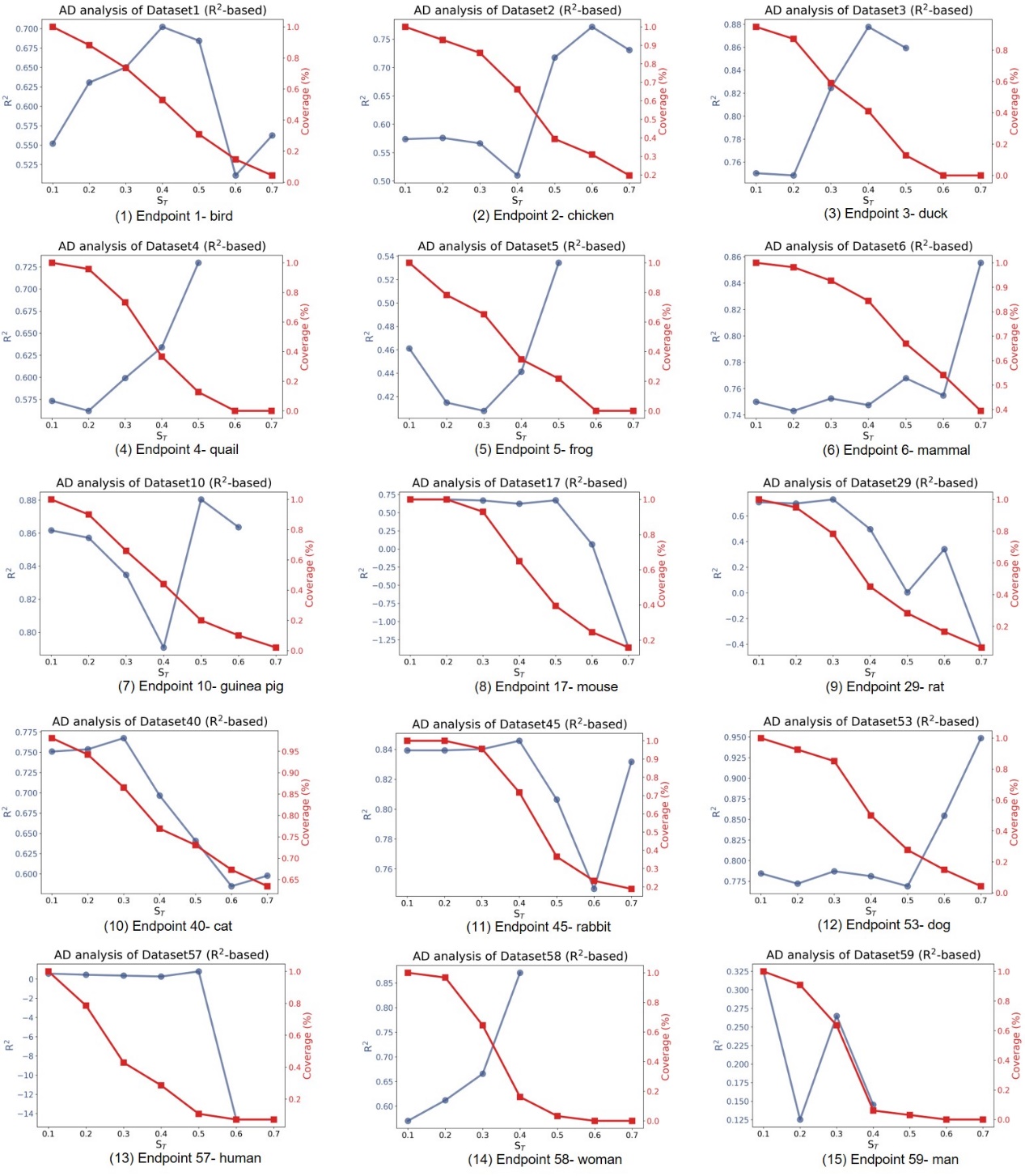


**Fig G1**. Performance metrics (R²) of samples in AD under varying thresholds. The x-axis represents the AD threshold S_T_. The left y-axis (blue lines) indicates metric values, while the right y-axis (red lines) denotes the proportion of extracted samples relative to the total.


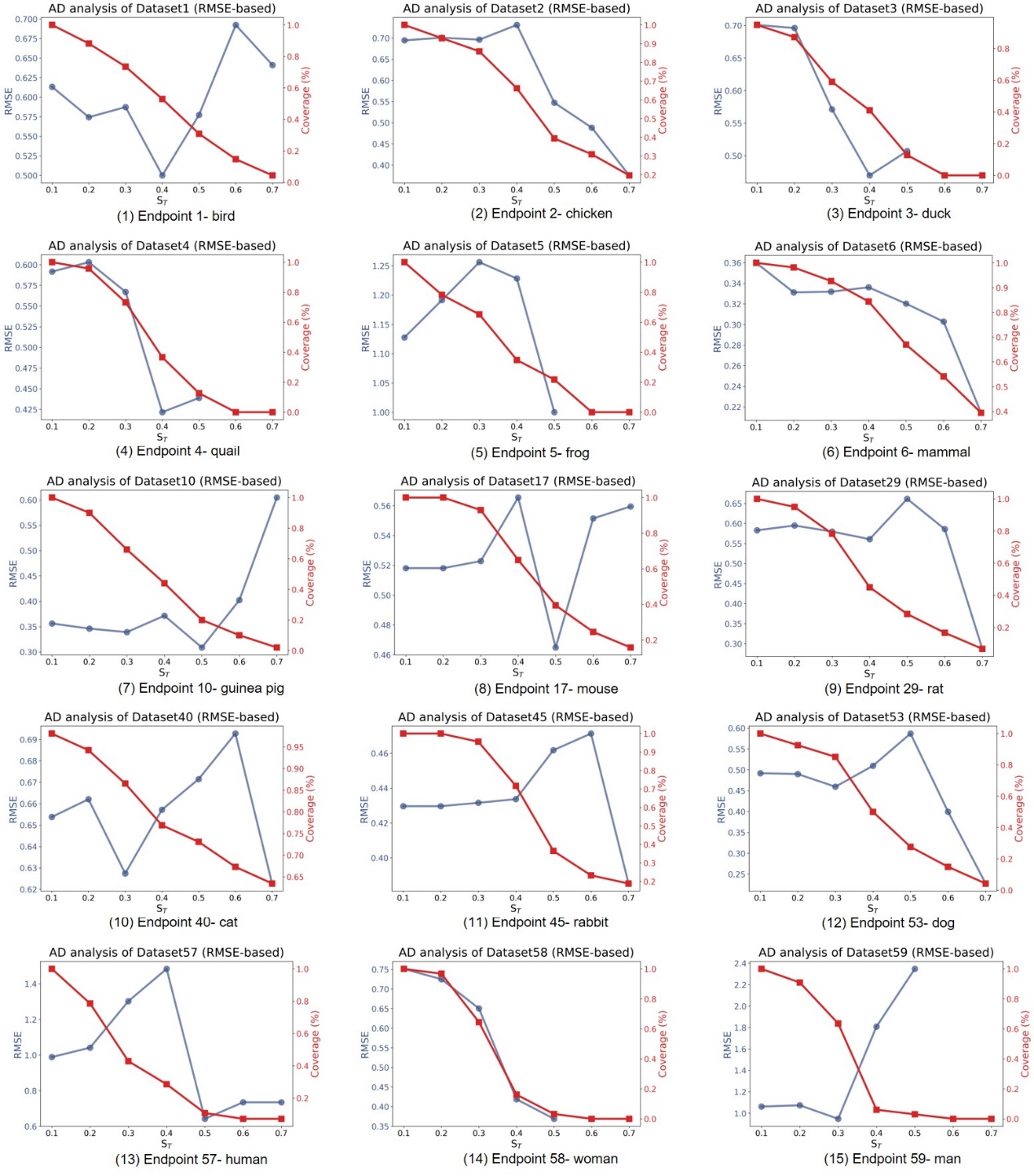


**Fig G2**. Performance metrics (RMSE) of samples in AD under varying thresholds.

1. **Uncertainty quantification**

Uncertainty Quantification (UQ) mainly addresses some critical challenges in high-dimensional dynamical systems and mathematical systems. In the current research field of Machine Learning (ML), there are very few studies related to the UQ of deep forest, and there is a lack of general tools for UQ of ML predictions. Apart from some methods that can directly provide confidence intervals of predictions, such as linear and polynomial models or Bayesian neural networks, most machine learning methods are unable to provide confidence intervals related to predictions. In response to the concerns raised by the reviewers, we conducted the UQ analysis from the following two aspects.

1. In ML, uncertainty can be simply classified into data uncertainty and model uncertainty. Data uncertainty is caused by the inherent and inevitable noise in the data, and it cannot be reduced by collecting more data. Our research data comes from existing literatures, based on which many published studies have been directly proposed. To maintain fair performance comparisons with published studies, we do not analyze data uncertainty.
2. Regarding model uncertainty, some studies have pointed out that "In deep ensembles methods, model uncertainty is often estimated by looking at an ensemble of trained models, the spread of predictions obtained from different models is then used as an estimate of model uncertainty". The proposed method is based on the modification of the deep forest framework. Its output layer is the ensemble of multiple forests, which can be described as the fusion of the ensemble of decision trees. Therefore, we can calculate the Prediction Standard Deviation (PSB) of all decision trees on the output layer in the testing set, and representing the average of all decision trees’ PSBs as the model's uncertainty. On 59 toxicity endpoints, we plotted the trend of PSB changes (Figure G3). A higher PSB indicates that the prediction uncertainty of the model at that toxicity endpoint is higher.

In Fig G3, the y-axis represents the PSB, and the x-axis corresponds to all toxicity endpoints. The yellow bars indicate the toxicity endpoints with larger PSBs, and the numbers above represent the size of these endpoints. It can be observed that the endpoints with larger PSB are primarily caused by small training samples. We can use the prediction results ± standard deviation to reflect the fluctuation range of the predictions. For example, when the R^2^ performance is higher and the standard deviation is smaller, it indicates that the prediction reliability of the model is higher.


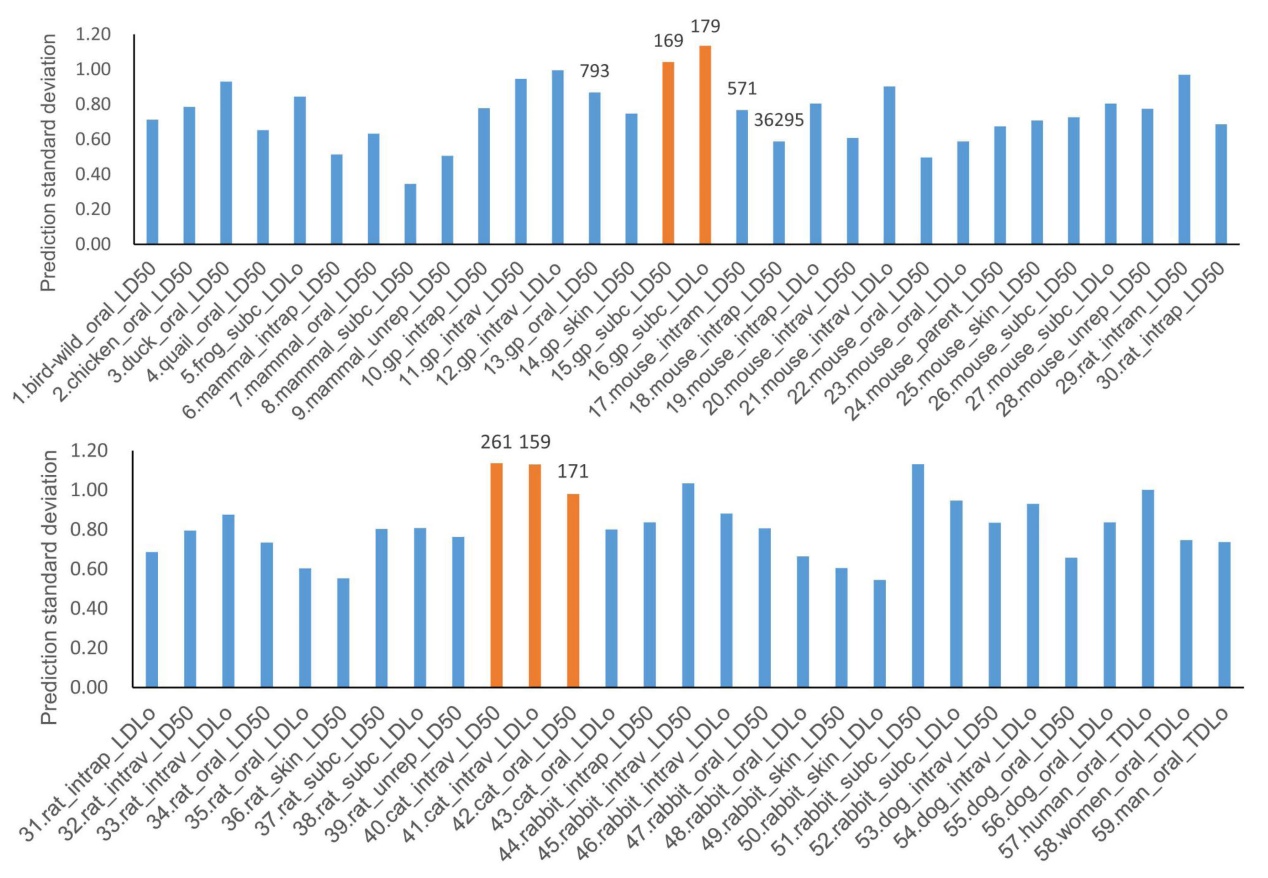


**Fig G3**. Predicted standard deviation (PSB) for all toxicity endpoints. The x-axis represents the toxicity endpoint. The y-axis indicates PSB, the yellow bars represent the toxicity endpoints with relatively large PSBs. The number above the bar indicates the number of samples corresponding to the endpoint.

#### Section H. Supplementary experiments during the third revision process

1. **Performance comparison of ensemble learning methods under single-task and multi-task paradigms**

| **Table H1.** Comparison of modeling performance of ensemble learning methods in single-task and multi-task paradigms (under Avalon feature space). | | | | |
| --- | --- | --- | --- | --- |
| ID | Model | R^2^ | RMSE | R^2^ growth by MT |
| 1 | ST-XGBoost | 0.28 | 0.83 | / |
| 2 | ST-LightGBM | 0.31 | 0.81 | / |
| 3 | ST-CatBoost | 0.38 | 0.77 | / |
| 4 | ST-Bagging | 0.32 | 0.80 | / |
| 5 | ST-Random Forest | 0.38 | 0.77 | / |
| 6 | MT-XGBoost | 0.39 | 0.76 | 0.11 ↑ |
| 7 | MT-LightGBM | 0.28 | 0.83 | 0.03 ↓ |
| 8 | MT-CatBoost | 0.38 | 0.77 | 0.00 = |
| 9 | MT-Bagging | 0.44 | 0.72 | 0.12 ↑ |
| 10 | MT-Random Forest | 0.46 | 0.71 | 0.08 ↑ |
| 11 | CFF-AT | 0.61 | 0.61 | / |

Note: ST refers to single-task, while MT refers to multi-task.

According to the reviewer's suggestion, we selected three serial ensemble methods (XGBoost, LightGBM, CatBoost) and two parallel ensemble methods (Bagging, Random Forest) to perform single-task and multi-task modeling, respectively. Under the single-task paradigm, the algorithm models and predicts for each toxicity endpoint separately. Under the multi-task paradigm, the training sets of all toxicity endpoints are combined for training the model, and then separate predictions are made for each toxicity endpoint's testing set. Table H1 reports the results of 10 runs of 5-fold cross-validation. From items 6 to 10 of Table H1, it can be seen that the performance of most ensemble learning methods in the multi-task paradigm is superior to or equal to that in the single-task paradigm (except for LightGBM), but is much lower than that of the proposed method (CFF-AT).

1. **The impact of different distance metrics on model performance**

| **Table H2.** Comparison of modeling performance among different distance metrics | | | |
| --- | --- | --- | --- |
| ID | Model | R^2^ | RMSE |
| 1 | CFF-AT with linear discrepancy | 0.60 | 0.62 |
| 2 | CFF-AT with frechet distance | 0.61 | 0.61 |
| 3 | CFF-AT with covariance distance | 0.61 | 0.61 |

| **Table H3.** Comparison of distance rankings between paired toxicity endpoints under three matrix metrics. | | | | | |
| --- | --- | --- | --- | --- | --- |
| Metrics | Ep1 vs Ep1 | Ep1 vs Ep2 | Ep1 vs Ep3 | Ep1 vs Ep4 | Ep1 vs Ep5 |
| LD | 0.0000 (1) | 22.513 (5) | 21.748 (4) | 11.769 (2) | 20.468 (3) |
| FD | 0.0000 (1) | 55.583 (4) | 49.229 (3) | 41.545 (2) | 74.241 (5) |
| CD | 0.0000 (1) | 0.0093 (4) | 0.0088 (3) | 0.0062 (2) | 0.0111 (5) |

Note: LD refers to linear discrepancy, FD refers to frechet distance, CV refers to covariance distance, Ep refers to toxicity endpoint.

First, we analyze the impact of different distance metrics on the model performance. We select three commonly used evaluation metrics for calculating the distance of pairwise matrixes from the field of transfer learning, such as Linear Discrepancy (LD), Frechet Distance (FD), and Covariance Distance (CD). From the results in Table H2, it can be seen that the performance of the proposed algorithm is relatively close when using different distance metrics, where the LD has slightly lower performance.

Second, we analyze the difference in distance ranking between toxicity endpoints under different evaluation metrics. Taking Table H3 as an example, the values in the table represent the distance relationships between the first toxicity endpoint and the top five toxicity endpoints. Since the calculated values under different evaluation metrics cannot be directly compared, we marked the distance ranking in parentheses. The smallest distance is represented by 1, the second smallest by 2, and so on. Taking the first column as an example (Ep1 vs Ep1), the distance between the first toxicity endpoint and itself is generally 0, so it ranks first. Combining the results of Table H2 and H3, it can be speculated that under the guidance of FD and CD, the ranking relationship between toxicity endpoints is consistent, resulting in similar modeling results. The ranking situation of LD is slightly different from that of FD and CD, which may be caused by the imprecise ranking of LD, leading to a slight decline in model performance.

Third, considering the modeling performance and the theoretical complexity of distance metrics, we selected the theoretically simple CD as the component of the proposed algorithm.
